# An NGS approach for the identification of precise homoeologous recombination sites between A and C genomes in *Brassica* genus

**DOI:** 10.1270/jsbbs.23090

**Published:** 2024-08-29

**Authors:** Tenta Segawa, Riki Kumazawa, Muluneh Tamiru-Oli, Tetsuyuki Hanano, Makishi Hara, Minami Nishikawa, Sorachi Saiga, Marina Takata, Masaki Ito, Tomohiro Imamura, Hiroki Takagi

**Affiliations:** 1 Ishikawa Prefectural University, 1-308 Suematsu, Nonoichi, Ishikawa 921-8836, Japan; 2 Department of Animal, Plant and Soil Sciences, La Trobe University, 5 Ring Road, Bundoora, VIC 3086, Australia; 3 Kanazawa University, School of Biological Science and Technology, College of Science and Engineering, Kakuma-machi, Kanazawa, Ishikawa 920-1192, Japan

**Keywords:** next-generation sequencing, *Brassica rapa*, *Brassica napus*, homoeologous recombination, allopolyploid

## Abstract

The introgression of heterologous genomes through interspecific hybridization offers a great opportunity to expand the gene pool of crops, thereby broadening the traits that can be targeted for improvement. The introgression of C genomic regions carrying desirable traits from *Brassica napus* (AACC) into the diploid *B. rapa* (AA) via homoeologous recombination (HR) has been commonly used. However, the precise identification of HR sites remains a significant challenge, limiting the practical application of genome introgression via HR in breeding programs. Here, we developed an indicator named ‘Dosage-score’ from the coverage depth of next-generation sequencing reads. Then, Dosage-score analysis applied to both in BC_1_F_1_ individuals obtained by backcrossing *B. rapa* to F_1_ progeny (*B. rapa* × *B. napus*) and in the parental lines, and successfully identified the precise HR sites resulting from F_1_ meiosis as well as those that were native in the parental *B. napus* genome. Additionally, we introgressed the C6 segment from HR identified by Dosage-score analysis into *B. rapa* genome background, revealing gene expression on the added segment without noticeable phenotypic change. The identification of HR by Dosage-score analysis will contribute to the expansion of the gene pool for breeding by introgression of heterologous genomes in *Brassica* crops.

## Introduction

A consequence of domestication and crop breeding has been the reduction of the genetic diversity present in modern crops (Doebley *et al.* 2006). Expanding the genetic pool of cultivated species remains a crucial challenge in breeding programs aiming to develop crops with desirable traits. Various methods are employed to enrich the genetic pool of crops: introducing new mutations via artificial mutagenesis, introducing genes from different species through transgenic techniques, and introducing novel genomes by homoeologous recombination (HR) via interspecific crossing.

Artificial mutagenesis using chemical mutagens such as ethyl methanesulfonate and N-methyl-N-nitrosourea or radiation using gamma rays and heavy-ion beams is rapid and efficient (Oladosu *et al.* 2016). However, the variation induced by a single mutagenesis is limited compared to the breadth of natural variations resulting from spontaneous mutations followed by long periods of environmental adaptation and artificial selection. Additionally, most artificial mutations tend to result in loss of gene function, limiting their utility for trait improvement. Genetic transformation can overcome interspecies barriers and allow the introduction of genes of interest into plants from other plant species or animals and bacteria (Altpeter *et al.* 2016). However, this is only possible in a handful of crop species for which transformation protocols are currently available. Also, the cultivation of crops improved via genetic transformation (commonly referred to as genetically modified crops), and those intended for direct human consumption, is still prohibited in most parts of the world (Marone *et al.* 2023).

HR between chromosomes originating from closely related plant species provides a promising opportunity to introduce genomic regions harboring genes associated with diverse traits that can be deployed in crop breeding programs (Leal-Bertioli *et al.* 2018, Mason and Wendel 2020, Shea *et al.* 2018, Zhang *et al.* 2017). An added advantage of this approach is that the cultivation of crops improved via HR is not subjected to any restriction. Despite the promising potential of HR in plant breeding, our understanding of its remains limited. Hence, there is a demand for understanding HR mechanisms and developing efficient methods to introduce HR, potentially expanding its application to a wider diversity of plant species.

*Brassica napus* (AACC), an allotetraploid species cultivated mainly for extraction of edible oil, is believed to have originated from the hybridization between *B. rapa* (AA) and *B. oleracea* (CC) ~7,500 years ago (Chalhoub *et al.* 2014, U 1935). Despite this hybrid origin, the A and C chromosomes occasionally pair during meiosis, resulting in HR in some *B. napus* cultivars (Higgins *et al.* 2021). If the *B. napus* lines carrying the A and C chromosomes/segments exchanged via HR are identified, continuous crossing of such lines to *B. rapa* and *B. oleracea* could provide an opportunity to introgress such chromosomal segments into genomes these two species. Consequently, HR in *B. napus* could lead to the expansion of the gene pools of *B. rapa* and *B. oleracea*, facilitating the introduction of novel traits for crop improvement.

The identification of HR regions has conventionally been time consuming and labor-intensive. HR between the *B. napus* A and C chromosomes are commonly identified using fluorescent labeling through techniques such as fluorescence *in situ* hybridization (FISH) and genomic *in situ* hybridization (GISH) (Higgins *et al.* 2021). While these visualization-based methods can detect HR across the entire genome, they fall short when it comes to identifying the precise HR sites. This in turn makes it difficult to confirm whether the target genes on the exchanged chromosome segment have indeed been inherited during the breeding process. Linkage analysis using DNA markers has also been applied to successfully identify rough HR regions between A and C genomes in doubled haploid *B. napus* lines (Udall *et al.* 2005). This technique is also time-consuming and labor-intensive as it requires the development of DNA markers across the entire genome. In particular, designing DNA markers that can distinguish the homoeologous regions between A and C genome is not a trivial task (Saiga *et al.* 2023). Taken together, the precise identification of HR sites has not been successfully achieved using currently available techniques. Consequently, a new or an improved technique that allows the accurate and efficient identification of HR sites is urgently needed to improve the effectiveness and speed of crop breeding via homoeologous genome introgression.

Recent progress in next-generation sequencing (NGS) technologies have enabled the chromosome-level assembly of many genomes (Le Nguyen *et al.* 2019). Within the *Brassica* genus, genome assemblies have been generated and made publicly available for several species and varieties (Kang *et al.* 2021, Paritosh *et al.* 2020, https://doi.org/10.1101/2022.10.13.512038). These include Chiifu-401-42 and Z1 in *B. rapa*, HDEM and OX-heart_923 in *B. oleracea*, and Darmor-bzh and ZS11 in *B. napus* (Belser *et al.* 2018, Guo *et al.* 2021, Rousseau-Gueutin *et al.* 2020, Sun *et al.* 2017, Wang *et al.* 2011). These reference genome sequences have greatly facilitated the re-sequencing and analysis of genomes of several individuals, making the comparison of genome structures more affordable and accessible.

An NGS-based method known as IntroMap has been developed for identifying HR based on coverage ratio of mapped NGS reads (Shea *et al.* 2017). IntroMap requires the generation of homozygous genomes via techniques such as doubled haploid and chromosome segment substitution lines, limiting its application to heterozygous genomes. Consequently, there is a clear need for the development of more versatile NGS-based methods capable of detecting HR across a wider range of genomes. In this study, we aimed to establish an NGS-based strategy to identify HR sites between the A and C chromosomes of *B. napus* and between progenies resulting from the cross between *B. napus* and *B. rapa* accurately and efficiently. To this end, we developed an indicator we named as ‘Dosage-score’, which represents the genomic dosage across the genome calculated from the coverage depth of sequence reads derived from next-generation sequencing. We then applied Dosage-score based analysis to parental lines and BC_1_F_1_ progeny obtained by backcrossing an F_1_ hybrid (*B. rapa* × *B. napus*) to *B. rapa* to successfully identify the precise HR sites. Furthermore, we developed substitution lines in *B. rapa* background carrying a C chromosome segment derived via exchange with A chromosome, demonstrating the potential application of this approach for improving traits of interest in *B. rapa*.

## Materials and Methods

### Plant materials

All the *B. rapa* cv. ‘CHOY SUM EX CHINA3’ and the 14 *B. napus* cultivars in this study were obtained from the National Agriculture and Food Research Organization (NARO) Genebank of Japan (https://www.gene.affrc.go.jp/index_en.php) (Supplemental Table 1).

### Flow cytometry estimation of nuclear DNA content

Nuclei were isolated by chopping leaves with a razor blade in a Nuclei Extraction Buffer provided in the CyStain UV Precise P kit (Sysmex). The mixture was then filtered through a 30 μm mesh, and the nuclei were stained with DAPI by adding Staining Buffer from the same kit. After a 10-minute incubation, the samples were analyzed using a CyFlow Ploidy Analyzer (Sysmex), following the manufacturer’s instructions.

### The generation of NGS data and a reference genome sequence for Dosage-score analysis

Illumina short reads previously generated for three *B. rapa* and four *B. oleracea* cultivars were downloaded from the NCBI Sequence Read Archive (SRA) using SRA-tools (https://github.com/ncbi/sra-tools.git) (Supplemental Table 2). The “NapusRef” used as reference sequence was prepared by concatenating *B. rapa* “Chiif v4.0 (http://brassicadb.cn/#/Download/) (Wang *et al.* 2011)” and *B. oleracea* “OX-herat-923 (https://www.ncbi.nlm.nih.gov/assembly/GCA_018177695.1/) (Guo *et al.* 2021)”. Because “OX-herat-923” also contained contigs that could not be anchored into chromosomes, such contigs were removed before “OX-herat-923” was concatenated. For whole-genome resequencing of *B. napus* cv. ‘HANNA’, *B. rapa* cv. ‘CHOY SUM EX CHINA3’, their F_1_ progeny, seven BC_1_F_1_ individuals, the 13 additional *B. napus* cultivars and a single BC_2_F_1_ individual, DNA samples were extracted using the DNeasy Plant Mini Kit (QIAGEN). Illumina sequencing libraries were prepared with the NEBnext Ultra II FS DNA Library Prep Kit (NEB), and sequenced on Illumina NovaSeq or HiSeqX platforms (Supplemental Table 3). Alignment of short reads was carried out using BWA ver. 0.7.17 (Li and Durbin 2009, https://arxiv.org/abs/1303.3997) and the conversion of aligned reads between SAM and BAM files were carried out using SAMtools ver. 1.16.1 (Li *et al.* 2009). Among the aligned file, the short reads from PCR duplicates and those showing mapping quality value of <60 were excluded.

### The filtering steps used to increase accuracy of Dosage-score analysis

In the repeat filter step, the repeat sequences of “Chiifu ver. 4.0” and “OX-heart-923” were identified with RepeatModeler version 2.0.3 and RepeatMasker version 4.0.6. Subsequently, the identified repeat sequences were excluded from the analysis with custom scripts. For the multi- and non-homoeologous filter step, the homologous regions of “Chiifu ver. 4.0” and “OX-heart-923” were detected using GetTwoGenomeSyn.pl with the “-MappingBin minimap2 -MinLenA 5000 -MinLenB 5000” options (He *et al.* 2023). Then, among the regions showing homology between the A and C genome, the regions showing homology with multiple regions of homoeologous genome were identified. Regions showing no homology with homoeologous genome were also detected. Both these regions were excluded from further analysis. In the ancestral diploid filter step, the sequence reads from four *B. rapa* and four *B. oleracea* cultivars (Supplemental Table 2) were first separately aligned to “NapusRef”. On the A genome, regions that failed to achieve a coverage depth > 0 for all *B. rapa* cultivars and coverage depth = 0 for all *B. oleracea* cultivars were also removed from the analysis. Similarly, on the C genome, regions failing to achieve a coverage depth > 0 for all *B. oleracea* cultivars and coverage depth = 0 for all *B. rapa* cultivars were also removed.

### Calculating Dosage-score

The steps for calculating Dosage-score were detailed in the result section. In *B. napus* cultivars, the median values of the average coverage depth in 2 Mb intervals on both A and C chromosomes were used to determine the average coverage depth of A and C chromosomes, respectively. In F_1_, BC_1_F_1_ and BC_2_F_1_, the median of the average coverage depth of only A genome was used to calculate the average coverage of the entire genome.

### Pipeline for filtering step and calculating Dosage-score

We have developed a pipeline in Python for all the filtering steps and the calculation of the Dosage-score. It is available from GitHub (https://github.com/SegawaTenta/Dosage-score). This pipline can be easily installed through the Bioconda platform (Grüning *et al.* 2018).

### Identifying homoeologous region between the A and C genomes

Homoeologous region between A and C chromosomes in “NapusRef” were defined by GetTwoGenomeSyn.pl with the options “-MappingBin minimap2 -MinLenA 5000 -MinLenB 5000” (He *et al.* 2023). Among the regions showing homoeologous relations, those regions that showed homology with more than two regions were excluded using custom scripts for reducing the complexity caused by homology of centromeric regions and transposable elements. Finally, we generated a graph using NGenomeSyn ver. 1.41 to visualize the results.

### qPCR with gDNA for confirming dosage in a genomic region

We estimated relative genome dosage by qPCR with gDNA. The gDNA was extracted using the DNA Suisui-P kit (Rizo) and then subjected to PCR with the KAPA SYBR FAST qPCR Master Mix (2X) Kit (KAPA BIOSYSTEMS). The PCR was performed on the StepOnePlus Real-Time PCR System (Applied Biosystems), which enables real-time monitoring of fluorescence from the PCR products. The PCR conditions were: an initial step at 95°C for 10 minutes, followed by 40 cycles of 95°C for 15 seconds and 60°C for 60 seconds, with a final step at 95°C for 15 seconds, 60°C for 60 seconds and 95°C for 15 seconds.

### Confirming phenotype and transcriptome in BC_2_F_2_

BC_2_F_2_ individuals were cultivated in growth chamber under constant temperature (22°C), photoperiod (14/10 h light/dark) and CO_2_ concentration (400–600 ppm). BC_2_F_2_ seeds were sown in a cell tray (4.05 cm × 4.05 cm), and two-week old seedlings were transplanted into 9 cm individual plastic pots and grown for an additional month to confirm their phenotype.

For transcriptome analysis by RNA-seq, RNA samples were extracted from the tip (approximately 2 cm) of fully expanded leaves of two-week-old seedlings. Total RNA was extracted using the RNeasy Plant Mini Kit (QIAGEN) and was then subjected to DNase treatment to remove contaminating DNA. RNA-seq libraries were constructed using the NEBNext Ultra II RNA Library Prep Kit for Illumina (NEB). The libraries were then sequenced on the Illumina Hiseq and NovaSeq platform (Supplemental Table 3).

To increase the accuracy of gene prediction on “NapusRef”, we initially assembled short reads from all RNA-seq samples using Trinity ver. 2.13.2 (Grabherr *et al.* 2011). These assembled contigs were then aligned to “NapusRef” using Minimap2 ver. 2.17 (Li 2018) with the ‘-ax’ option for aligning long reads. Following this, we predicted the genes on “NapusRef” based on the locations of the aligned contigs by Stringtie ver. 2.0.6 (Pertea *et al.* 2015) with the ‘-L’ option for adapting the long reads alignment. Next, we aligned each RNA-seq sample to “NapusRef” by Hisat2 ver. 2.1.0 (Sirén *et al.* 2014) and counted the number of reads on predicted genes by Featurecount (subread ver. 2.0.0) (Liao *et al.* 2014). Finally, differentially expressed genes were defined by comparing the read counts between two groups using DESeq2 ver. 1.10.1 (Love *et al.* 2014).

## Results

### The principle of Dosage-score analysis

In this study, we developed an indicator that we named ‘Dosage-score’ and applied it for estimating the genome structure of BC_1_F_1_ progeny carrying heterologous genomes from *B. rapa* and *B. napus*. Dosage-score is a normalized coverage depth of NGS reads that are aligned to a reference genome of interest and represents genome dosage at a specific genomic region.

**Fig. 1.**
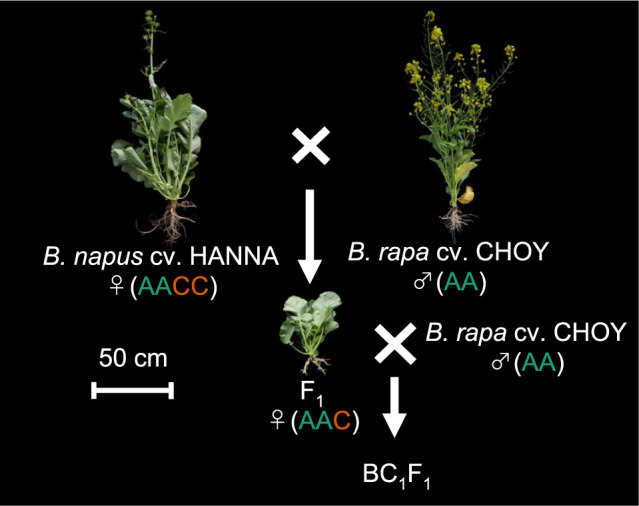
The *Brassica* species and their progeny used in this study. A schematic illustrating the crossed made to generate the BC_1_F_1_ progeny used in this study. Of the 63 BC_1_F_1_ individuals generated, seven were subjected to Dosage-score analysis (Fig. 3, Supplemental Fig. 3), while all 63 individuals were analyzed by PCR to confirm presence/absence of the C chromosomes (Fig. 6). Genomes of the parental lines and their F_1_ hybrid are shown in parenthesis.

To generate the BC_1_F_1_ progeny used in this study, we crossed the *B. rapa* cv. ‘CHOY SUM EX CHINA3’ (hereafter ‘CHOY’) and the *B. napus* cv. ‘HANNA’ and obtained F_1_ hybrid. We then crossed the F_1_ hybrid to ‘CHOY’ and generated a BC_1_F_1_ population consisting of 63 individuals (Fig. 1). The nuclear DNA contents of ‘CHOY’, ‘HANNA’, their F_1_ hybrid and two BC_1_F_1_ plants were compared using flow cytometry analysis. As expected, G_1_ peak position of ‘HANNA’ was about double compared to that of ‘CHOY’ on the histogram of relative fluorescence intensity, whereas the G_1_ peak of their F_1_ progeny was halfway between the ‘HANNA’ and ‘CHOY’ peaks (Supplemental Fig. 1). These observations confirmed that ‘CHOY’ (*B. rapa*) and ‘HANNA’ (*B. napus*) are diploid (AA) and tetraploid (AACC), respectively, and their F_1_ hybrid is triploid (AAC). Two BC_1_F_1_ individuals, BC_1_F_1_-E and BC_1_F_1_-G, independently analyzed had G_1_ peaks at two different positions, both of which were lower than the G_1_ peak position of the F_1_ hybrid. This suggested different levels of C genome introgression from *B. napus* into the BC_1_F_1_ individuals.

**Fig. 2.**
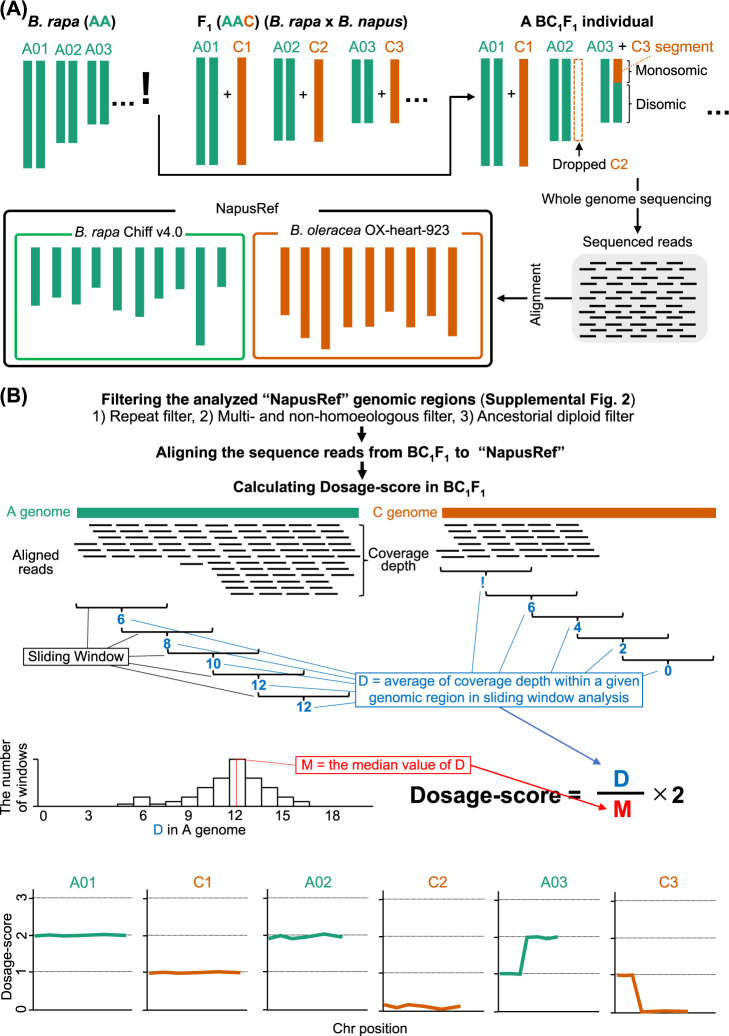
Flow chart of the steps involved in Dosage-score based analysis. (A) The cross between *B. rapa* and an F_1_ progeny (*B. rapa* × *B. napus*) generates segregating BC_1_F_1_ progeny. NGS reads obtained for the BC_1_F_1_ progeny are then aligned to NapusRef, a reference genome sequence of *B. naups* developed by concatenating the A and C genomes of *B. rapa* and *B. oleracea*, respectively. (B) Following alignment of NGS reads, three filter steps are used to increase the accuracy of Dosage-score values, based on which the genome structure of individual genotypes are determined.

A flowchart depicting the steps involved in the analysis of genome structure using Dosage-score is presented (Fig. 2, Supplemental Fig. 2). First, we prepared a pseudo reference genome sequence of *B. napus*, “NapusRef”, by concatenating the A genome of *B. rapa* “Chiff ver. 4.0” and the C genome of *B. oleracea* “OX-heart-923” (Guo *et al.* 2021, Wang *et al.* 2011) (Fig. 2A). *B. napus* (AACC) is an allotetraploid hybrid that originated from *B. rapa* (AA) and *B. oleracea* (CC). Following alignment of the BC_1_F_1_ NGS short reads to the “NapusRef”, we applied three filter steps: repeat filter, multi- and non-homologous filter and ancestorial diploid filter to exclude the genomic regions that can result in erroneous coverage depth, thereby increasing accuracy of the Dosage-score calculation. At the repeat filter step, the genomic regions containing repeat sequences, such as the transposon sequences and centromere repeats, are identified by RepeatModeler2 and RepeatMasker and excluded from further analysis (Chen 2004, Flynn *et al.* 2020) (Supplemental Fig. 2A). The multi- and non-homoeologous filter step identifies and excludes the A genome regions that show no or multiple homologies with C genome regions and vice versa using GeTwoGenomeSyn.pl (He *et al.* 2023) (Supplemental Fig. 2B). This is followed by the ancestral diploid filter step at which the genomic regions showing misalignment and inconsistent alignment of NGS reads from multiple lines/cultivars of the diploid ancestral species are detected and excluded (Supplemental Fig. 2C).

After filtering, Dosage-score representing the genome dosage is calculated from the short read alignment data using the formula Dosage-score = (*D*/*M*) × 2; where *D* represents the average coverage depth of NGS reads at a specific genomic region in sliding window analysis and *M* is the median value of *D* for the disomic chromosomes. If a window in the sliding window analysis excludes over 95% of its region based on the filter criteria, it is excluded from the analysis. Dividing *D* by *M* across all genomic regions provides a normalized *D* value, which is then multiplied by 2, giving a genome dosage score of 2 to disomic genomic regions (Fig. 2B).

The A chromosomes of our BC_1_F_1_ plants (AA × AAC genomes) are expected to be in disomic state in the absence of HR with the C genome. As *M* in BC_1_F_1_ is calculated from all *D* values of A chromosomes, the Dosage-score in the disomic A chromosome regions will be ~2. The Dosage-score of C chromosome introgressed into BC_1_F_1_ from *B. napus* will be ~1, while a Dosage-score of ~0 represents the absence of C chromosome introgression. The chromosomal regions that experienced HR between the A and C chromosomes are identified based on shifts in Dosage-score by more than 1.

### Dosage-score analysis identifies HR regions in BC_1_F_1_ progeny

**Fig. 3.**
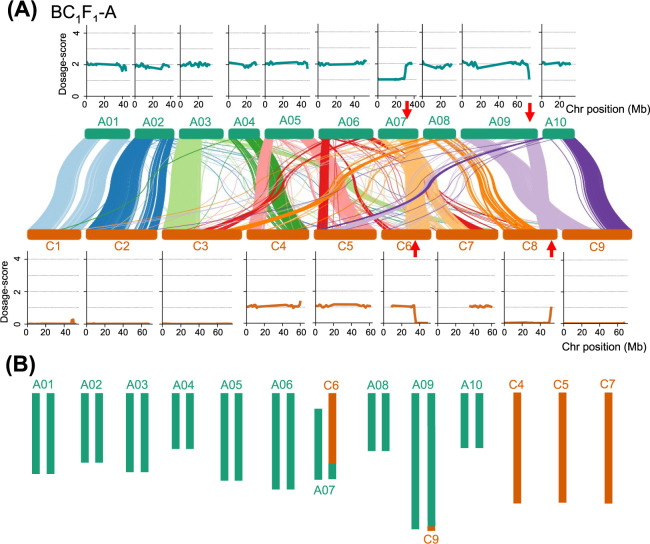
The Dosage-score based analysis in of BC_1_F_1_-A. (A) Dosage-score values of A (top panel) and C chromosomes (lower panel) of BC_1_F_1_-A. Dosage-score analysis was set window size = 2 Mb and step size = 500 kb. The homoeologous regions between the A and C genomes generated by NGenomeSyn were shown in the middle. Red arrows indicate the chromosomal regions that experienced shifts in Dosage-score values by over 0.5. (B) The genome structure of BC_1_F_1_-A predicted from the Dosage-score patterns and homoeologous relations shown in (A).

As a proof-of-principle, we applied the Dosage-score analysis to a single BC_1_F_1_ plant, BC_1_F_1_-A, and assessed the genome dosage for all chromosomes (A01–A10 and C1–C9) (Fig. 3). Our analysis revealed that BC_1_F_1_-A displayed Dosage-score of ~1 across the entire C4, C5 and C7 chromosomes, suggesting that it inherited these chromosomes from ‘HANNA’ (*B. napus*). We determined that the Dosage-scores of C1, C2, C3 and C9 were close to 0, indicating that these chromosomes were absent in BC_1_F_1_-A genome (Fig. 3A).

There was a notable reduction in the Dosage-score from 1 to 0 for C6 from ~35 Mb region, suggesting that the first 35 Mb region of C6 was present in the BC_1_F_1_-A genome in a monosomic state. Conversely, the Dosage-score of C8 increased from 0 to 1 from the 51 Mb region, indicating that the posterior region of C8:51 Mb was present in the BC_1_F_1_-A genome in a monosomic state (Fig. 3A). Additionally, reductions in Dosage-score from the 2 to 1 were observed for the first 30 Mb region of A07 and from 72.5 Mb region of A09 (Fig. 3A). For the BC_1_F_1_ line analyzed here, which was obtained from the cross between F_1_ (AAC) and ‘CHOY’ (AA) (Fig. 1), the genome dosage of A should be 2 with an expected Dosage-score of 2 for the A genome. The two genomic regions of A07 and A09 with Dosage-scores of ~1 should correspond to the homoeologous regions of C6 and C8 with Dosage-scores of ~1, respectively (Fig. 3A). Therefore, we hypothesized that HR events occurred at A07:30 × C6:35 Mb and A09:72.5 × C8:51 Mb regions. These events would result in the recombination of the anterior part of A07:30 Mb with the posterior part of C6:35 Mb, and the anterior part of A09:72.5 Mb with the posterior part of C8:51 Mb, respectively (Fig. 3B).

### Applying Dosage-score based analysis to parental lines, multiple BC_1_F_1_ progeny and diverse *B. napus* cultivars

**Fig. 4.**
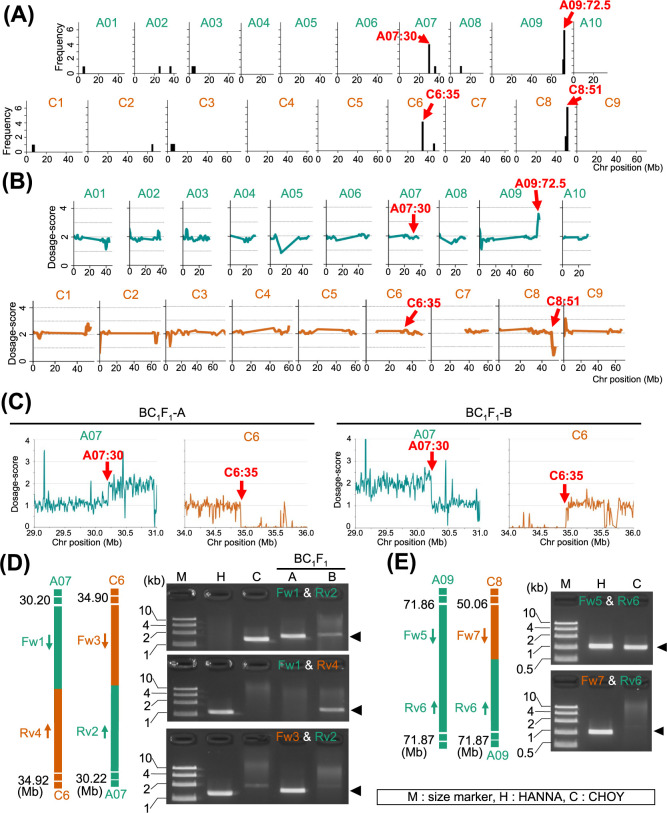
Identification of native HR in *B. napus* cv. ‘HANNA’. (A) Frequency distribution of individuals with chromosomal locations showing significant Dosage-score changes among the seven BC_1_F_1_ individuals analyzed. These locations were identified following alignment of NGS short reads to “NapsRef”. (B) The Dosage-score across the entire genome for ‘HANNA’. Dosage-score analysis was set window size = 2 Mb and step size = 500 kb. (C) Dosage-scores determined using a narrower sliding window size (10 kb) and step size (5 kb) within the A07:29–31 and C6:34–36 Mb chromosomal regions two BC_1_F_1_ individuals: BC_1_F_1_-A and BC_1_F_1_-B. Red arrowheads represent HR sites (D) Verification of the reciprocal type and (E) nonreciprocal type native HR of ‘HANNA’ in A07:30.2 × C6:34.9 Mb and A09:71.8 × C8:50.0 Mb chromosome pair by PCR, respectively. Black arrowheads show the expected PCR product sizes.

To investigate the frequency of HR between the A and C chromosomes, we applied Dosage-score analysis to six additional BC_1_F_1_ individuals and assessed their genome structures (Fig. 4A, Supplemental Fig. 3). Similar to BC_1_F_1_-A, the additional BC_1_F_1_ lines analyzed inherited either the entire or segments of C chromosomes as revealed by the frequency regions showing Dosage-scores of greater than 1 (Fig. 4A). For all the seven BC_1_F_1_ individuals analyzed, we counted the number of genomic regions where Dosage-score values changed by more than 0.5 and defined these as HR regions. We then categorized these regions into two types as unique and common. The unique HR regions are those that are only detected in a single BC_1_F_1_ individual, and these HR types are assumed to be the result of HR between the A and C genomes during F_1_ meiosis. In contrast, the common HR types are shared by two or more BC_1_F_1_ individuals such as the ones detected at A07:30, A09:72.5, C6:35 and C8:51 Mb regions (Figs. 3A, 4A). We hypothesized that these common HR types are native to the parental *B. napus* ‘HANNA’.

To test the hypothesis that the HR types commonly detected in BC_1_F_1_ progeny are native HR regions of ‘HANNA’, we applied Dosage-score analysis to ‘HANNA’ (Fig. 4B). ‘HANNA’ exhibited Dosage-score changes at A09:72.5 and C8:51 Mb regions, which corresponded to two of the four common HR regions detected in BC_1_F_1_ progeny (Fig. 4A). No notable Dosage-score changes were detected in ‘HANNA’ at the two remaining common HR regions of BC_1_F_1_, A07:30 and C6:35 Mb (Fig. 4B). We further classified the HR regions in ‘HANNA’ into two as reciprocal and nonreciprocal. The reciprocal HR involves exchanges of segments between the A and C chromosomes without changes in Dosage-score across the entire chromosomes. Conversely, the nonreciprocal types result both in the substitution of chromosomal segments between the A and C chromosomes and loss of one of the substituted genomic regions, leading to doubling of the substituted region. Consequently, the Dosage-score for chromosomes exhibiting reciprocal type native HR should be 2 across the entire chromosome, while those displaying nonreciprocal type native HR should be 0 in one chromosome and 4 in its homoeologous counterpart. Based on the Dosage-score values, the commonly detected HR type of A07:30 and C6:35 Mb denote locations of reciprocal type native HR, while regions A09:72.5 and C8:51 Mb indicate locations of nonreciprocal type native HR in ‘HANNA’. The positions A07:30 and C6:35 Mb also corresponded to regions known for reciprocal native HR in some *B. napus* cultivars (Udall *et al.* 2005).

To pinpoint the accurate HR site within the A07:30 and C6:35 Mb region of ‘HANNA’ that was delineated using a sliding window size of 2 Mb, we applied sliding window analysis with a smaller window size (10 kb) to two lines: BC_1_F_1_-A and BC_1_F_1_-B (Fig. 4C). Despite the noise in Dosage-score peaks, we identified a common region with notable Dosage-score changes in both BC_1_F_1_-A and BC_1_F_1_-B, specifically between the A07:30.205–30.220 and C6:34.905–34.915 Mb genomic regions. We defined this as a notable region based on a change in Dosage-score value greater than 0.5 from the expected score (2 for the A genome, 0 or 1 for the C genome). Within the A07:30.205–30.220 region, we investigated the border positions showing two-fold change in coverage depth of aligned reads in both lines. Additionally, within the C6:34.905–34.915 Mb region, we investigated the border positions where the coverage depth changed to ~0. As the results, we identified the changing coverage depth pattern at A07:30.215–30.216 and C6:34.909–34.910 Mb regions (Supplemental Fig. 4A), suggesting that these genomic regions represent the reciprocal type of native HR sites in ‘HANNA’. To verify this finding, we performed PCR using a primer pair designed at the flanking region of the detected HR sites. As expected, PCR successfully amplified the products from the recombinant allele in ‘HANNA’, while no amplification was detected in ‘CHOY’ using the same primer pair (Fig. 4D, Supplemental Fig. 4B).

Dosage-score analysis of the nonreciprocal type HR sites at A09:72.5 and C8:51 Mb using the smaller 10-kb sliding window size revealed two distinct sub-regions within each chromosome region based on Dosage-score changes: A09:71.8 and A09:72.9 Mb for A09:72.5 Mb region and C8:50.0 and C8:51.3 Mb for the C8:51 Mb region (Supplemental Fig. 5A). By detecting the transition points at which coverage depth change more than doubled, we pinpointed the precise HR sites at A09:71.864–71.865, A09:72.981–72.982, C8:50.064–50.065, and C8:51.315–51.316 Mb for A09:71.8, A09:72.9, C8:50.0, and C8:51.3 Mb, respectively.

In ‘HANNA’, the Dosage-score at the nonreciprocal type HR sites between A09:71.8–72.9 Mb in the A genome was about 4, and about 0 for those HR sites between C8:50.0–51.3 Mb, suggesting that the C genome between C8:50.06–51.31 Mb is absent in ‘HANNA’. Additionally, the Dosage-score at the posterior region of A09:72.9 Mb was ~3, likely due to the introgression of one C genome posterior region of C8:51.3 Mb (Supplemental Fig. 5B). The genomic structure of ‘HANNA’ at A09:71.8, A09:72.9, C8:50.0 and C8:51.3 Mb regions was validated using PCR with primer pairs flanking regions of each HR site (Fig. 4E, Supplemental Fig. 5C–5E). Similarly, we verified the genomic dosage in the region between A09:71.8 and C8:50.0 Mb by qPCR of gDNA (Supplemental Fig. 5F).

**Fig. 5.**
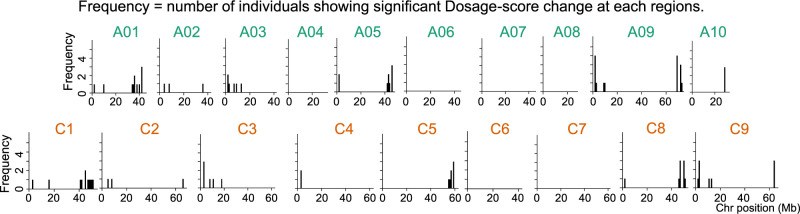
Identification of nonreciprocal type HR sites in diverse *B. napus* cultivars. Frequency distribution of individuals showing significant Dosage-score changes across each A and C chromosomes. Frequency = number of cultivars showing significant dosage-score changes at a specific chromosomal region. These chromosomal regions/positions were identified following alignment of 13 *B. napus* NGS short reads to “NapsRef”.

We further applied Dosage-score analysis to diverse cultivars of *B. napus* and successfully identified HR events, demonstrating the wider applicability of the method. Among the 13 *B. napus* cultivars analyzed, we identified 47 positions that exhibited significant Dosage-score changes, suggesting nonreciprocal type HR at these positions (Fig. 5, Supplemental Fig. 6).

### Efficiency of C chromosome introgressions into the A genome of *B. rapa* through HR

**Fig. 6.**
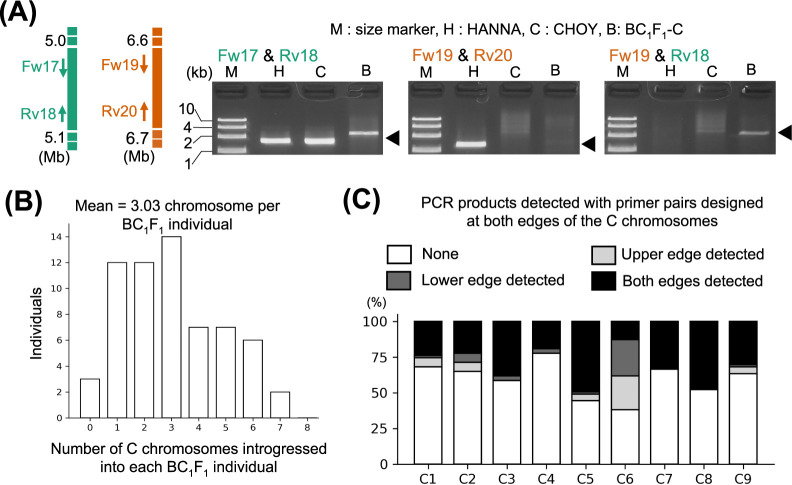
Identification of HR sites that occurred at the F_1_ meiotic stage. (A) Verification of the HR site between the A01:5.0 Mb and C1:6.6 Mb chromosome regions in BC_1_F_1_-C. Black arrowheads indicate the expected sizes of PCR products amplified using the primer pairs shown above the gel images. (B) Frequency distribution of the number of C chromosomes (excluding C6) introduced into each BC_1_F_1_ individual. The presence of each chromosome was confirmed by PCR using primer pairs designed at both ends of each C chromosome in a total of 63 BC_1_F_1_ individuals. The count reflects individuals wherein at least one edge of the C chromosome was detected. (C) The proportion of C chromosomes introduced into each of the 63 BC_1_F_1_ individuals. The presence of each C chromosome was confirmed by PCR using the same primers used in (B).

As shown above (Fig. 4A), our Dosage-score analysis detected unique HR sites in some BC_1_F_1_ individuals that were predicted to have occurred during F_1_ meiosis. Indeed, we confirmed the unique HR site located at A01:5.07 and C01:6.65 Mb regions in BC_1_F_1_-C by PCR, with a similar strategy used to determine the native HR site for ‘HANNA’ (Fig. 6A, Supplemental Fig. 7). Although Dosage-score detected a total of 5 unique HR sites within the seven BC_1_F_1_ individuals analyzed, the question about the frequency of unique HR sites for each chromosome remains.

To evaluate the efficiency of C chromosome introgression into the A genome, we further investigated the proportion of C chromosome segments introgressed across 63 BC_1_F_1_ individuals by PCR with DNA markers specifically designed at both termini of each C chromosome (Fig. 6B, 6C, Supplemental Table 4). If introgression was detected at both ends of the C chromosome by PCR, we inferred that the entire chromosome had been introgressed into BC_1_F_1_ progeny. Conversely, when only one end of the C chromosome was detected, we inferred that a segment of the C chromosome had been integrated into the homoeologous A chromosome by HR. After accounting for both entire and segmental introgression of C chromosomes and excluding the C6 chromosome exhibiting native HR from ‘HANNA’, we estimated that on average 3.03 C chromosomes were introduced per BC_1_F_1_ individual (Fig. 6B). This is low considering that four C chromosomes (half of eight C chromosomes, excluding C6) are expected to be inherited in BC_1_F_1_. On the other hands, the heritability of C chromosome was varied by depending on each chromosome. The average heritability of C chromosomes in BC_1_F_1_ was 28.3%, which is half of the expected ratio of 50% based on the assumption that C chromosome is randomly inherited by BC_1_F_1_ (AA + C) from F_1_ progeny (AAC) (Fig. 6C). About 49.2% of C5 and 47.6% of C8 were detected in BC_1_F_1_ progeny, suggesting that heritability of these chromosomes was similar to that of the A chromosomes in *B. rapa*. Conversely, the C4 exhibited a lower heritability at less than 20%, implying its resistance to introgression into A genome. Additionally, the estimated proportion of the introgression of anterior and posterior region of C6 was 36.5% and 38.1%, respectively. Although PCR products at both termini of the C6 were detected in 12.7% BC_1_F_1_ progeny, this is expected to be a result of both the anterior and posterior region of C6 from reciprocal type HR inherited in a BC_1_F_1_ individual. On average, the proportion of segments of the remaining C chromosomes introgressed into BC_1_F_1_ progeny because of HR was 4.96%, with C2 showing the highest ratio at 12.7%. No HR was detected for C7 and C8.

To understand how the C chromosome is inherited from an F_1_ with an AAC genome, we assessed the meiotic pairing stage of the C chromosome in F_1_ through the SNP pattern in a specific BC_1_F_1_ individual, BC_1_F_1_-E. This individual exhibited a unique HR event between chromosomes A01 and C1, which are entirely syntenic. (Fig. 3A). Initially, we identified SNP positions between ‘CHOY’ and ‘HANNA’ on chromosome A01. Subsequently, we defined the genotype at each SNP position based on the frequency of SNP alleles in ‘NapusRef’. By integrating the results of the Dosage-score analysis with the SNP pattern, we deduced the genome structure of A01 and C1 chromosome in BC_1_F_1_-E. In this structure, BC_1_F_1_-E possessed a double recombinant chromosome: one between A01 of ‘CHOY’ and ‘HANNA’ at the 38.58 Mb region, and another between A01 from ‘HANNA’ and C1 from ‘HANNA’ due to HR at the 5.49 Mb region. The existence of this double recombinant chromosome in BC_1_F_1_-E suggests that trivalent chromosomes were formed during the meiosis of F_1_ (Supplemental Fig. 8).

### The effect of C6 downstream region from native HR of ‘HANNA’ on ‘CHOY’ genome

**Fig. 7.**
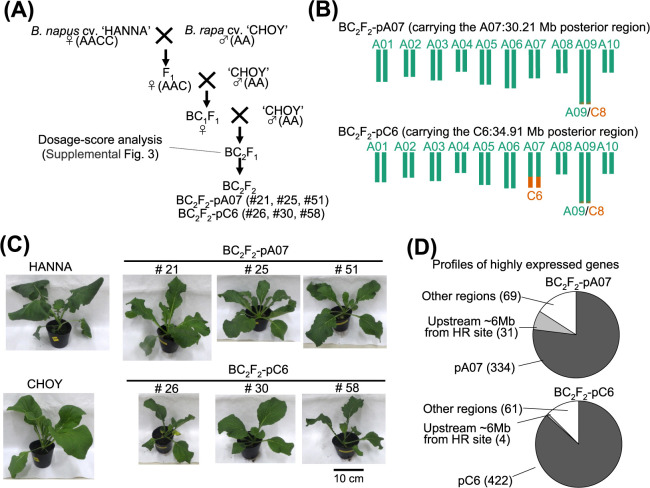
Effect of the C6:35 Mb chromosomal region introduced into *B. rapa*. (A) A schematic diagram showing the crosses made to develop the BC_2_F_2_ progeny segregating for the presence/absence of the pC6 region introduced from *B. napus* cv. ‘HANNA’. (B) The predicted genome structures of BC_2_F_2_-pA07 and BC_2_F_2_-pC6. (C) Phenotypes of one month old parental lines and BC_2_F_2_ genotypes grown under constant conditions (T° = 22°C, 14 h/10 hr light/dark photoperiod). (D) Gene expression profiles showing significant differences between BC_2_F_2_-pA07 and BC_2_F_2_-pC6 genotypes. Genes with a p-adjusted value in DESeq2 of less than 0.01 were defined as differentially expressed.

To evaluate the effects of the posterior region of C6:34.91 Mb (pC6) introgression in ‘CHOY’, which was exchanged with posterior region of A07:30.21 Mb (pA07) in ‘HANNA’ due to reciprocal type HR, we developed BC_2_F_2_ individuals by selfing of a BC_2_F_1_ individual harboring pC6 (Fig. 7A). We applied Dosage-score analysis to confirm that BC_2_F_1_ carried no C chromosomes, with the exception of pC6, and that the posterior region of C8:72.98 Mb in monosomic state (Supplemental Fig. 3). Accordingly, we considered that the BC_2_F_2_ population segregating for the presence/absence of pC6 should be suitable to evaluate the effect of pC6 on ‘CHOY’ (Fig. 7B). After one month of cultivation in a growth chamber under constant temperature (22°C) and photoperiod (14 hr/10 hr of light/dark), we visually compared the leaf morphology (including trichome density and leaf color and thickness) and flowering habits of BC_2_F_2_ lines carrying pC6 and pA07 (Fig. 7C). We observed no visually notable differences between the individuals with and without pC6 for the above traits even though the pC6 chromosome segment carried more than 527 genes. Furthermore, BC_2_F_2_ individuals carrying pC6, in place of pA07, successfully produced BC_2_F_3_ seeds through self-pollination. Taken together, this suggests that pC6 has minimal effect on both phenotypic traits and fertilization.

To confirm expressions of the genes encoded by pC6, we performed RNA-seq on BC_2_F_2_ individuals with and without pC6. In this analysis, we first mapped RNA-seq data from 3 BC_2_F_2_ individuals carrying pC6 and 3 BC_2_F_2_ individuals carrying pA07 to “NapusRef” to predict genes and their structure. We accordingly predicted 21,271 genes whose expression levels we compared between BC_2_F_2_ individuals carrying pC6 and pA07 using DEseq2 (Supplemental Table 5). As expected, the genes located on pA07 and pC6 exhibited higher expression in BC_2_F_2_ individuals carrying pA07 and pC6, respectively (Fig. 7D, Supplemental Fig. 9). This determined that the genes located within the pC6 region can express in the A genome background. Interestingly, 31 genes located within the 6 Mb upstream region of the HR site of A07:30.21 Mb showed higher expression in BC_2_F_2_ individuals carrying pA07, suggesting that the expression of these genes was repressed in BC_2_F_2_ individuals carrying pC6.

## Discussion

In this study, we successfully established an NGS-based analysis, Dosage-score, for estimating genome structure in BC_1_F_1_ lines obtained by backcrossing *B. rapa* with an F_1_ progeny generated from a cross between *B. rapa* and *B. napus*. We applied Dosage-score analysis to identify the precise HR sites that are both native to *B. napus* and those that originated from F_1_ meiosis.

Dosage-score has several benefits compared to the currently available approaches for identifying HR. First, Dosage-score is a more accurate and rapid method for identifying genome-wide HR sites compared with the conventional methods that include FISH, GISH and DNA marker-based linkage analysis. While the *Brassica* 60K Infinium SNP array has been recently applied to HR analysis, to our knowledge, it has not pinpointed actual HR sites within a 100 kb interval, as this depends on the positions of the SNPs (Clarke *et al.* 2016, Katche *et al.* 2023). Therefore, these conventional methods, including SNP arrays, are not entirely suitable for breeding programs that necessitate detailed information on gene combinations transferred from homoeologous genomes. Second, application of the NGS-based method for HR identification, called IntroMap, requires homozygous genomes such as chromosome substitution and doubled haploid lines. Whereas Dosage-score analysis can be applied to plants with heterozygous genomes, such as the BC_1_F_1_ progeny used in this study. This feature of Dosage-score analysis makes it suitable for use in breeding programs in which back crossed lines carrying heterozygous homoeologous genomes from different species are selected. Third, because Dosage-score analysis does not require high sequencing depth, sequencing cost will not limit the application of this method in breeding. In this study, we successfully identified HR sites with an average sequencing depth of about six. As the current decreasing cost of sequencing driven by advances in NGS techniques is also expected to continue, the application of NGS-based techniques such as Dosage-score become more affordable (Le Nguyen *et al.* 2019).

The efficiency and accuracy of Dosage-score for HR sites identification makes the method suitable for cataloging native HRs in diverse *B. napus* cultivars and in *B. napus* synthesized by hybridization between its diploid progenitors *B. rapa* and *B. oleracea*. In this study, we successfully identified 49 positions with native HRs across diverse 14 *B. napus* cultivars (Figs. 4B, 5). Except in ‘HANNA’, we only detected nonreciprocal type detected in these cultivars. Subsequent analysis in BC_1_F_1_ progeny is expected to reveal additional reciprocal type HRs, enriching the native HR catalog. If genes of agronomic importance are identified in the A and C genomic regions of *B. napus*, and a catalog with DNA markers specific to each HR site is developed, it allows efficient introgression of desirable genes from *B. napus* into *B. oleracea* and *B. rapa* without developing F_1_ hybrids (*B. rapa* × *B. oleracea*) and doubled haploid lines.

On the other hand, to increase the efficiency of introgression of C chromosome segment into the A genome via novel HR in F_1_, understanding the segregation of AAC chromosomes during meiosis in F_1_ and the differential inheritance rates among C chromosomes in F_1_ are crucial. The SNP pattern for the A01 and C1 chromosomes in the BC_1_F_1_-E progeny suggested the potential formation of trivalent chromosomes during F_1_ meiosis (Supplemental Fig. 8). If three homoeologous chromosomes (A01+A01+C1) segregate evenly during F_1_ meiosis, the expected gamete genotype segeregation in F_1_ would be A01A01:A01C1:A01:C1 = 1:2:2:1 in F_1_. This would suggest that the genotype in BC_1_F_1_ segregate A01A01A01:A01A01C1:A01A01:A01C1 = 1:2:2:1. However, no BC_1_F_1_ individuals were found carrying A01A01A01 and A01C1 chromosomes among the seven analyzed in BC_1_F_1_, despite the presence of BC_1_F_1_ individuals with A01A01C1 and A01A01. Furthermore, trisomy for other A chromosomes was not observed in BC_1_F_1_ plants. These findings suggest that the distribution of trivalent AAC chromosomes during F_1_ meiosis is biased, leading to an absence of gametes with complete length AA or C chromosomes. Additionally, these observations imply that replacing an entire A chromosome with its homoeologous C chromosome in *B. rapa* is challenging due to the absence of BC_1_F_1_ individuals with specific complete length C chromosomes replacing their homoeologous A chromosomes. Therefore, segmental substitution between A and C chromosomes via HR might be required the more feasible strategy for introgression of the desired C genomic regions into *B. rapa*, as demonstrated by our successful development of the BC_2_F_2_ carrying A07-pC6 chromosome. On the other hand, the differential inheritance rates among C chromosomes were observed in BC_1_F_1_ (Fig. 6C). The observed differential inheritance rates among C chromosomes in BC_1_F_1_ did not correspond with a previous study reported by Shea *et al.* (2018). In this study, we observed that the inheritance rate of chromosome C4 is the lowest. Contrastingly, Shea *et al.* (2018) did not observe a particularly low inheritance rate for chromosome C4 in their sample, instead identifying chromosome C6 as having the lowest rate. This variation in inheritance rates suggests that the specific crossing combinations used can affect the inheritance of each C chromosome. To determine factors influencing the efficiency of C chromosome inheritance, expanding the range of crossing combinations and investigating their inheritance rates is needed.

Additionally, determining the preferred locations and sequences for HR is crucial for boosting HR event frequency to effectively introgression of C genome regions. In this study, we described that the frequency of HR event was increased near chromosome edges in BC_1_F_1_ (Figs. 4A, 5) and the precise sequence occurred in an HR event from F_1_ was identified through Dosage-score analysis (Supplemental Fig. 7C). To understand HR preferences and potentially induce HR artificially, it is crucial to expand HR identifications using Dosage-score analysis and compare the sequences at these HR locations with normal recombination events within the A or C genome.

Development of the BC_2_F_2_ progeny carrying the A07-pC6 chromosome demonstrated the potential application of the approach for improving traits of interest in *B. rapa* via expression of the genes found on the pC6 chromosomal segment. Because there were no visible phenotypic differences between BC_2_F_2_-pC6 and BC_2_F_2_-pA07 plants, our finding suggested the possibility that functional traits, including biotic and abiotic stress tolerance, can be improved without introducing alterations to morphological traits. The 31 genes located within the 6 Mb upstream region of pC6 on A07 were repressed in BC_2_F_2_ lines homozygous for pC6. This repression is likely due to a cis regulatory enhancer element, not a trans element, affecting chromatin structure and the expression of closely located genes. Our understanding of how gene expression in chromosomal regions near HR sites is controlled has to improve in order to achieve targeted modifications of genes controlling traits of interest with no or minimum off-target effects.

In conclusion, several crop species of considerable economic importance that are produced and consumed worldwide are allopolyploids. These crops include coffee (*Coffea arabica*), strawberry (*Fragaria* × *ananassa*), wheat (*Triticum aestivum*), tobacco (*Nicotiana tabacum*) and cotton (*Gossypium hirsutum*). The Dosage-score based analysis detailed in this study can significantly contribute to our understanding of the genome structure, including the identification of native HRs, of these crops. The identified HRs could in turn greatly aid in breeding efforts of the diploid ancestors of these allopolyploid crops, effectively expanding their gene pool and contributing to the improvement of existing crops as well as the development of novel cultivars.

## Author Contribution Statement

TS developed the Dosage-score analysis and applied it to the BC_1_F_1_ generation. RK utilized the Dosage-score analysis for 13 *B. napus* cultivars. MT oversaw the study and wrote the paper. TH identified homoeologous recombination in *B. napus* cv. ‘HANNA’. MH and SS provided PCR support. MN created progeny from crosses between *B. rapa* and *B. napus*. MT also contributed to developing DNA markers for identifying homoeologous recombination. MI conducted flow cytometry analysis. TI provided overall supervision for the study. HT was responsible for designing and supervising the study, as well as wrote the paper. All authors played a role in writing the manuscript.

## Supplementary Material

Supplemental Figures

Supplemental Table 1

Supplemental Table 2

Supplemental Table 3

Supplemental Table 4

Supplemental Table 5
