# Supplementary material for: An NGS approach for the identification of precise homoeologous recombination sites between A and C genomes in Brassica genus
Source: Breed Sci. 2024 Aug 29;74(4):324–36. doi: 10.1270/jsbbs.23090 (PMC11769586; doi:10.1270/jsbbs.23090)
Supplement: Supplementary file 1 — Supplemental Figures [file 74_324_s1.pdf]

Segawa et al./Breeding Science
doi: 10.1270/jsbbs.23090

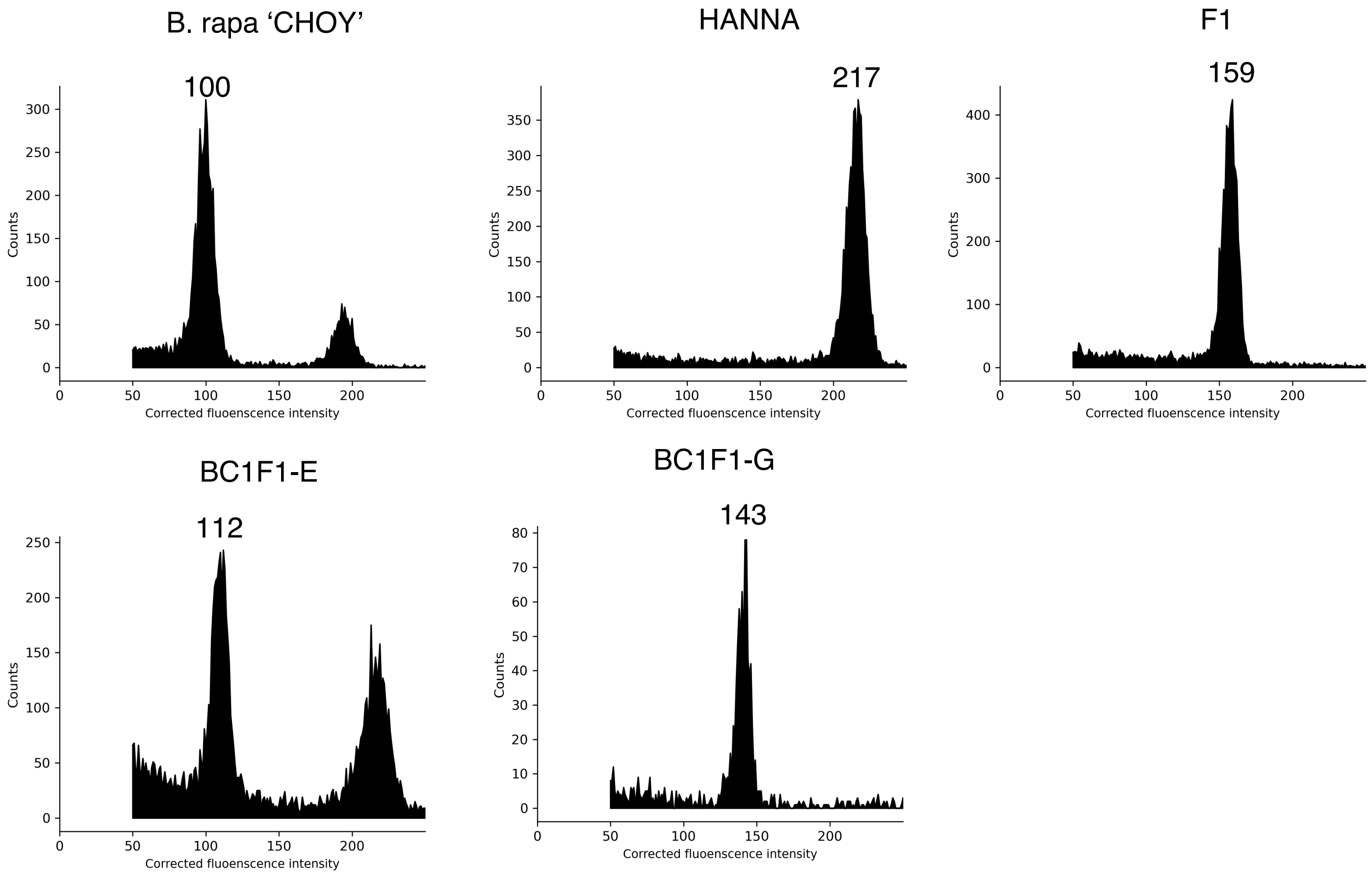

Supplemental Fig. 1. Flow cytometry-based estimation of nuclear DNA content.
The frequency distribution florescence intensity obtained by flow cytometry analysis of nuclei isolated from the parental lines, their F1 hybrid and BC1F1 progeny. The fluorescence intensity was corrected to a scale where 'CHOY' represented 100. The number on the graph correspond to the fluorescence intensity of the highest peak.

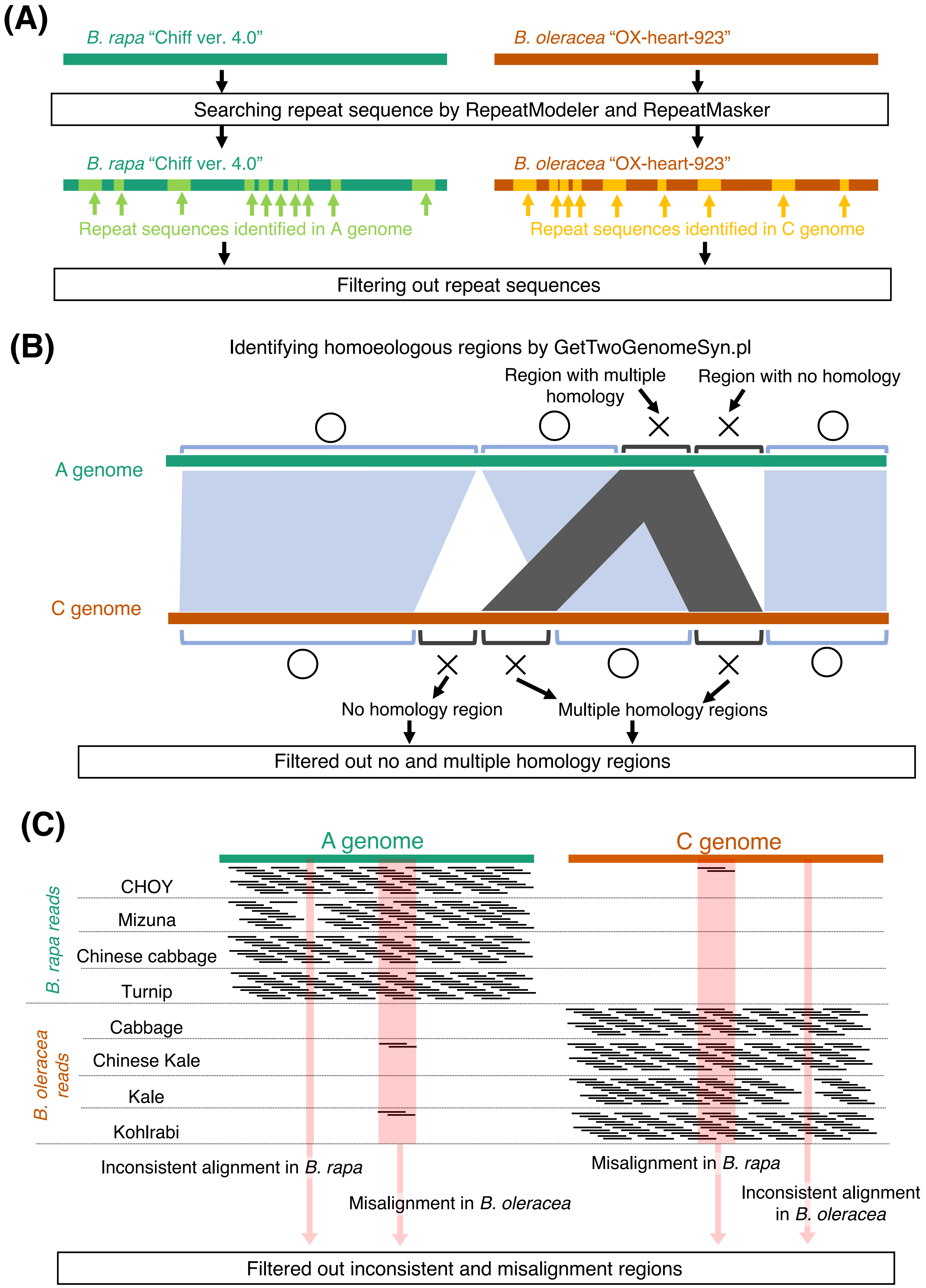
(A)
B. rapa "Chiff ver. 4.0"
B. oleracea "OX-heart-923"
Searching repeat sequence by RepeatModeler and RepeatMasker
B. rapa "Chiff ver. 4.0"
B. oleracea "OX-heart-923"
Repeat sequences identified in A genome
Repeat sequences identified in C genome
Filtering out repeat sequences
(B)
Identifying homoeologous regions by GetTwoGenomeSyn.pl
Region with multiple homology
Region with no homology
A genome
C genome
No homology region
Multiple homology regions
Filtered out no and multiple homology regions
(C)
A genome
C genome
B. rapa reads
CHOY
Mizuna
Chinese cabbage
Turnip
B. oleracea reads
Cabbage
Chinese Kale
Kale
Kohlrabi
Inconsistent alignment in B. rapa
Misalignment in B. oleracea
Misalignment in B. rapa
Inconsistent alignment in B. oleracea
Filtered out inconsistent and misalignment regions

Supplemental Fig. 2. Defining the genomic regions to be used for Dosage-score analysis.
Genomic regions containing (**A**) repeat and centromeric sequences and (**B**) no and multiple homology are identified and excluded from the analysis. Specifically, in this study, a 2 Mb interval containing centromeric sequences, which hit 70% of CentBr and PCRBr, was excluded. (**C**) Coverage depth-based selection relied on the aligned sequence reads from multiple *B. rapa* and *B. oleracea* cultivars.

F1

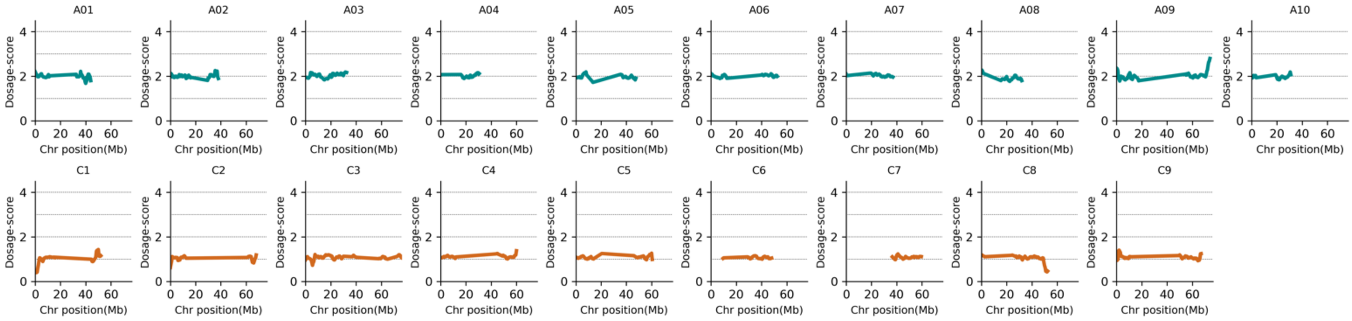

BC1F1-B

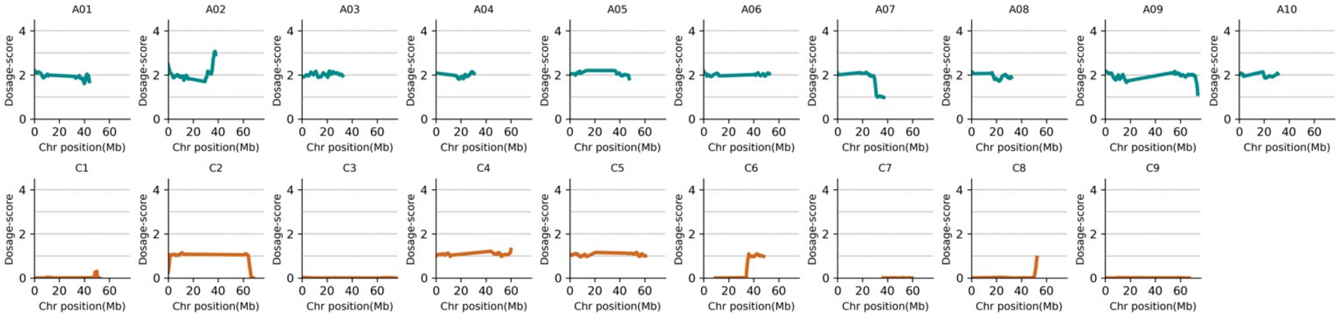

BC1F1-C

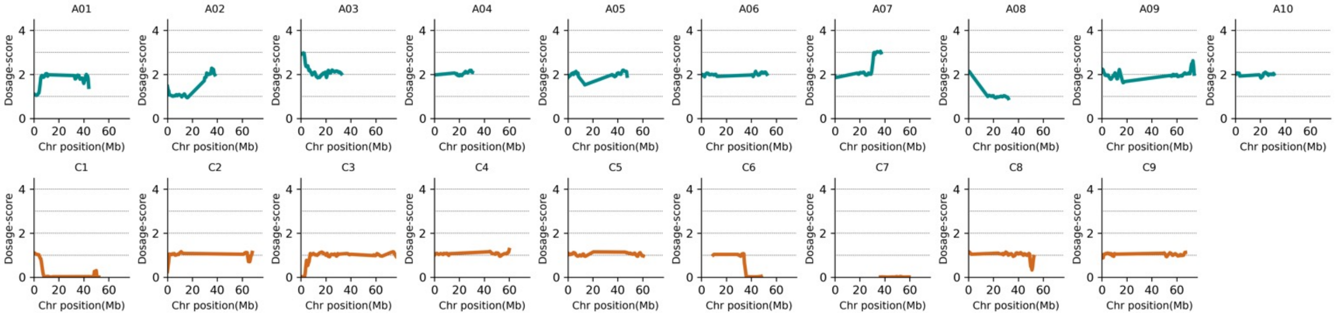

BC1F1-D

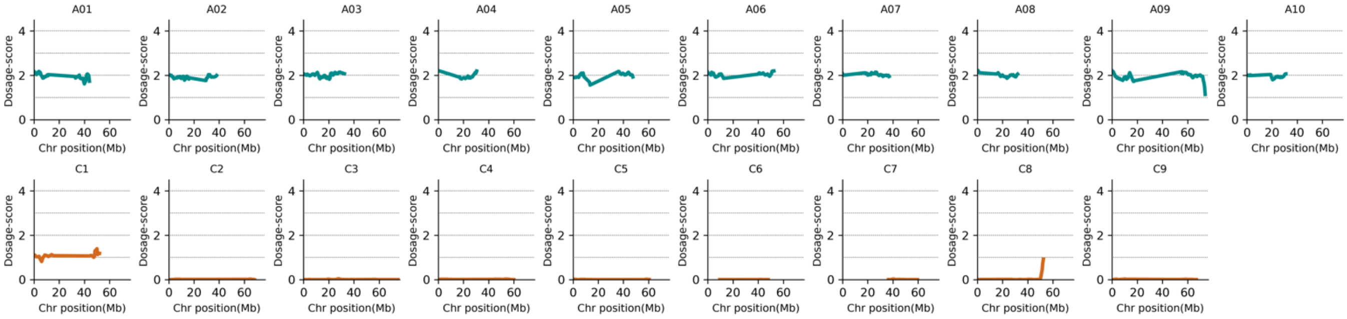

BC1F1-E

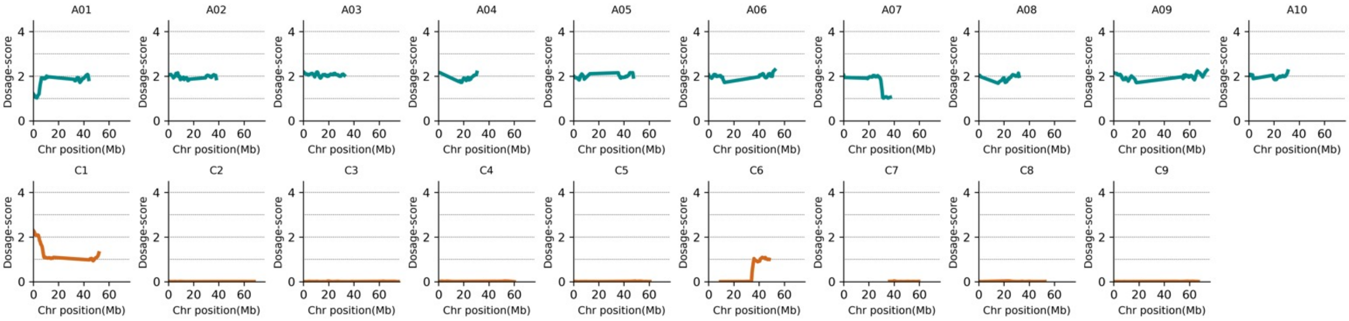

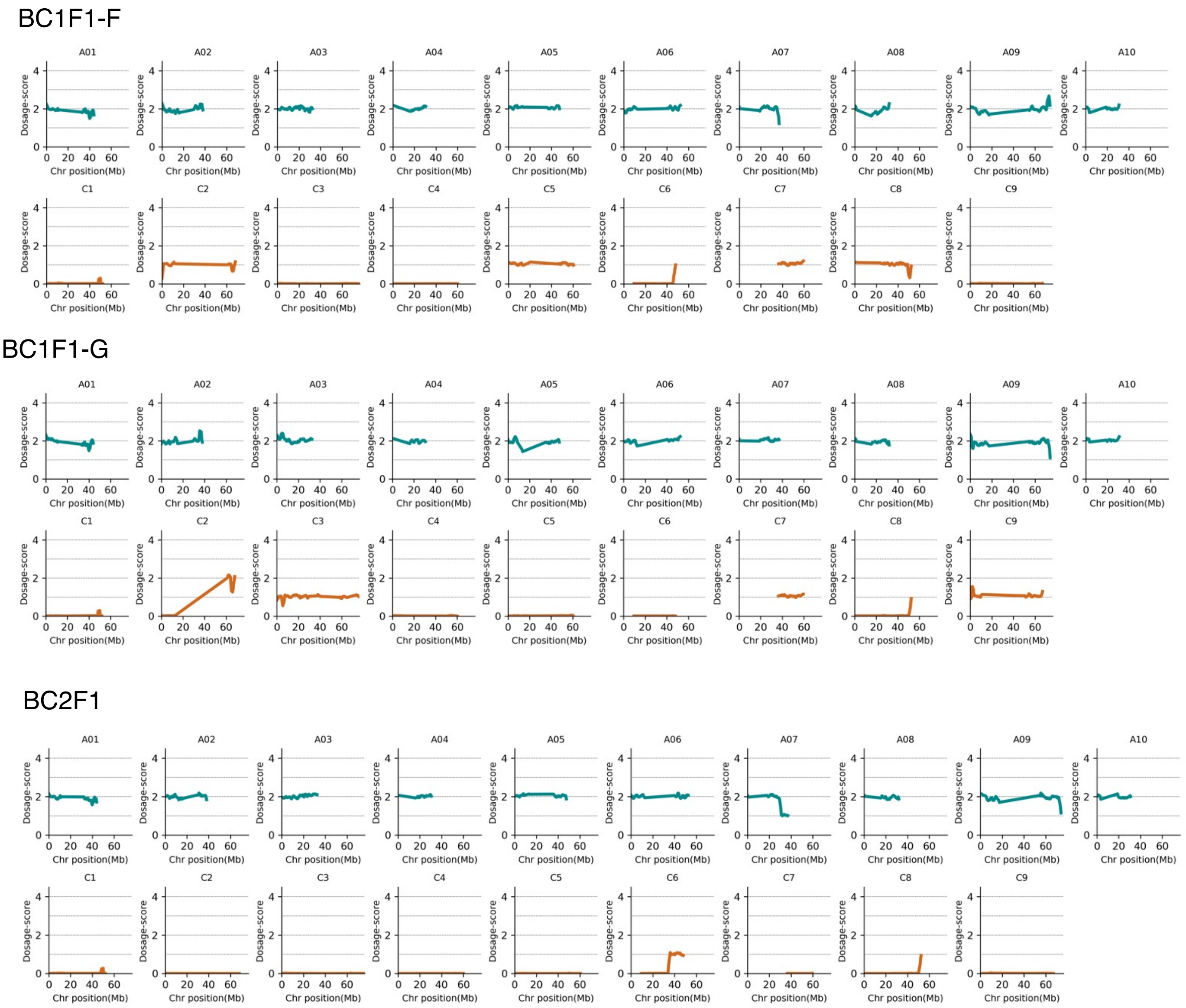

Supplemental Fig. 3. Dosage-score analysis in F1, BC1F1 and BC2F1 progeny.

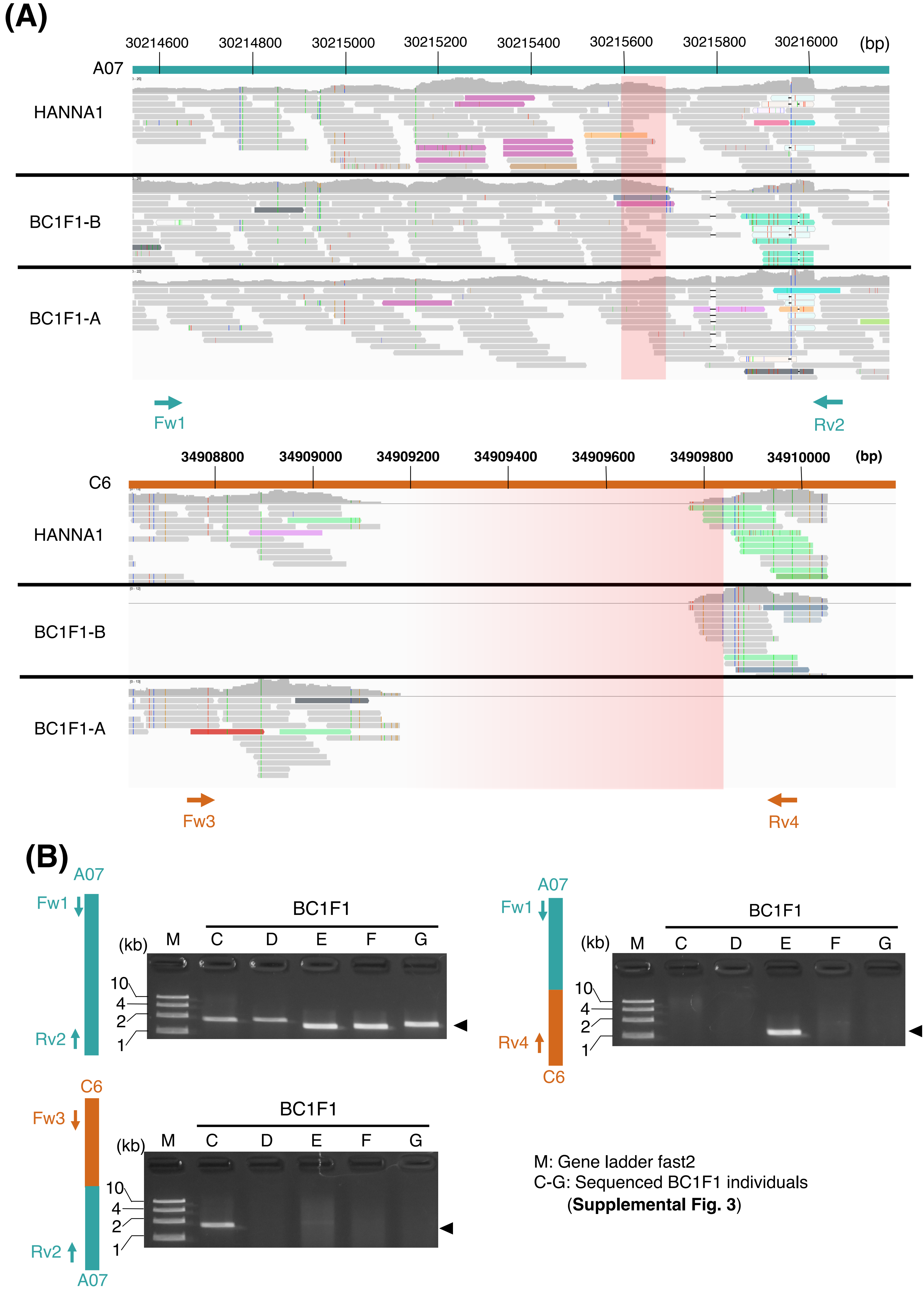
(A)
30214600
30214800
30215000
30215200
30215400
30215600
30215800
30216000
(bp)
A07
HANNA1
BC1F1-B
BC1F1-A
Fw1
Rv2
34908800
34909000
34909200
34909400
34909600
34909800
34910000
(bp)
C6
HANNA1
BC1F1-B
BC1F1-A
Fw3
Rv4
(B)
A07
Fw1
Rv2
BC1F1
(kb)
M
C
D
E
F
G
10
4
2
1
A07
Fw1
Rv4
C6
BC1F1
(kb)
M
C
D
E
F
G
10
4
2
1
C6
Fw3
Rv2
A07
BC1F1
(kb)
M
C
D
E
F
G
10
4
2
1
M: Gene ladder fast2
C-G: Sequenced BC1F1 individuals
(Supplemental Fig. 3)

(C)

C_A07 : CHOY amplified Fw1 and Rv2
H_C6A07 : HANNA amplified Fw3 and Rv2
H_A07C6 : HANNA amplified Fw1 and Rv4

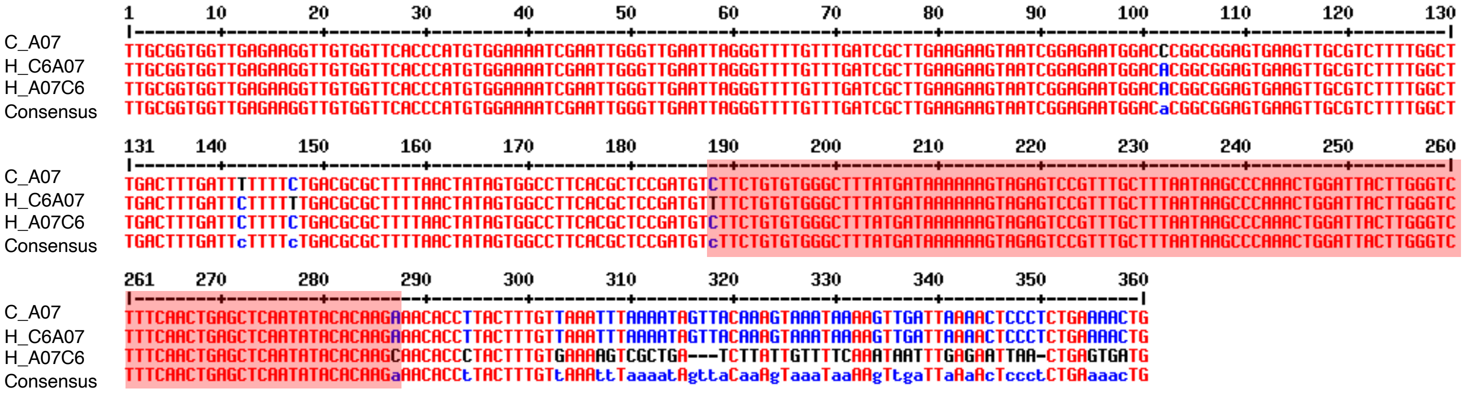

Supplemental Fig. 4. Identifying reciprocal type native HR sites in A07-C6.
(**A**) IGV images of HNNAH and BC1F1 progeny Illumina short reads aligned to the predicted A07 (top panel) and C6 (lower panel) HR sites. Sequence of the region shown in red was confirmed by Sanger sequencing. (**B**) Verification of reciprocal type native HR sites of ‘HANNA’ A07 x C6 chromosome pair by PCR. Black arrowheads indicate the expected PCR product sizes amplified by the primer pairs shown in the left of the gel images. (**D**) Comparison of sequences obtained by Sanger sequencing of PCR products (Fig. 4D) amplified using primers designed at HR sites in ‘HANNA’ and ‘CHOY’. The region highlighted in red represents the candidate HR site at a resolution of less than 100 bp interval.

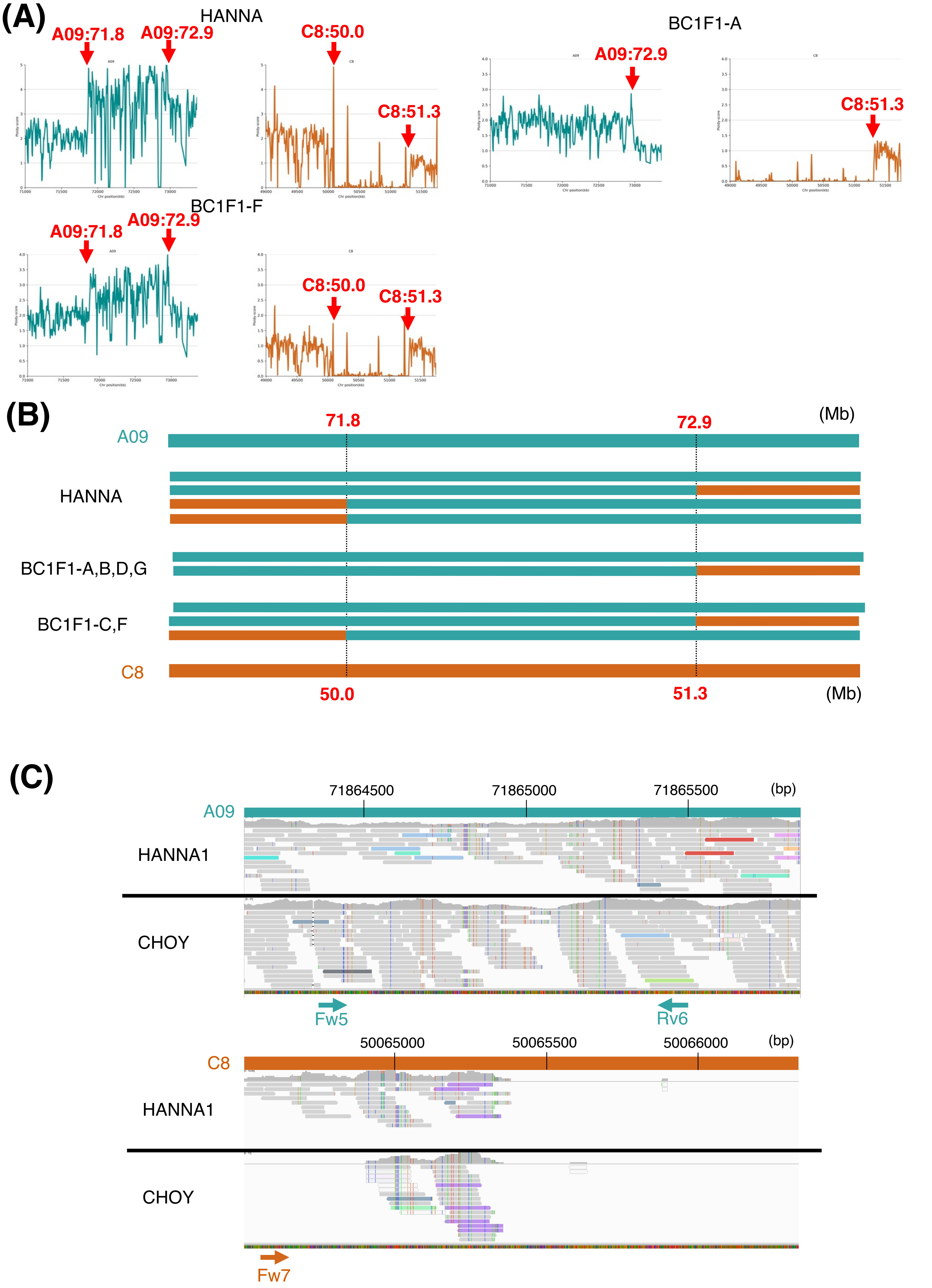

(A)
HANNA
A09:71.8
A09:72.9
C8:50.0
C8:51.3
BC1F1-A
A09:72.9
C8:51.3
BC1F1-F
A09:71.8
A09:72.9
C8:50.0
C8:51.3
(B)
A09
71.8
72.9
(Mb)
HANNA
BC1F1-A,B,D,G
BC1F1-C,F
C8
50.0
51.3
(Mb)
(C)
A09
71864500
71865000
71865500
(bp)
HANNA1
CHOY
Fw5
Rv6
C8
50065000
50065500
50066000
(bp)
HANNA1
CHOY
Fw7

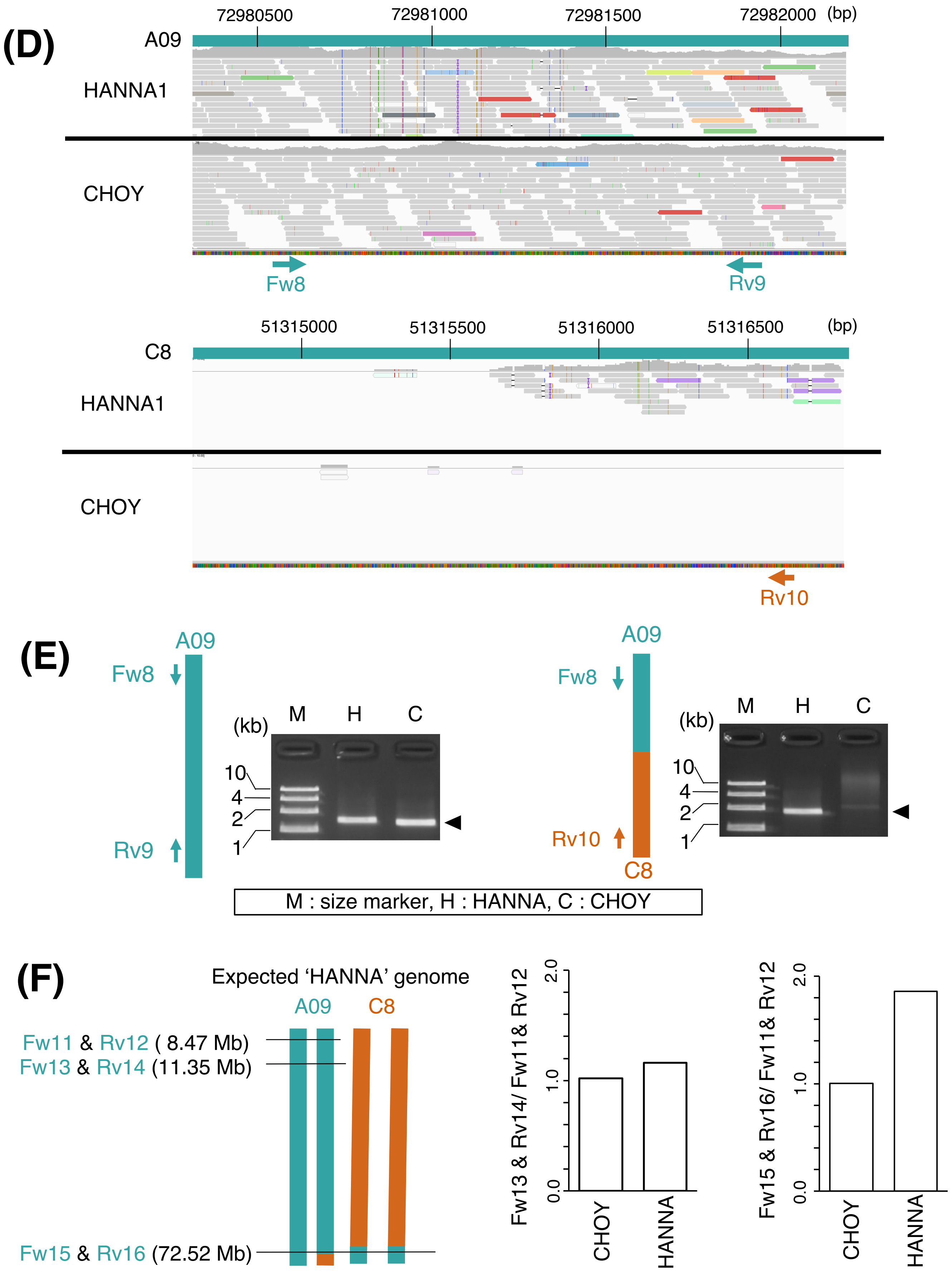

Supplemental Fig. 5. Identifying nonreciprocal type native HR site in A09-C8.
(A) Dosage-scores analysis on A09 and C8 on chromosomes of 'HANNA', BC1F1-A and BC1F1-F using a sliding window size of 10 kb and step size of 5 kb. (**B**) The genome structure estimated from the Dosage-score patterns and homoeologous relations presented in (A). (**C, D**) IGV images of the Illumina short reads aligned to the predicted HR sites of A09:71.8 and C8:50.0 (C) and A07:72.9 and C8.51.3 (D). (**E**)Verification of 'HANNA' nonreciprocal type native HR sites in C8:50.0 and C8.51.3 pair by PCR. Black arrowheads indicate the expected sizes of PCR products amplified using the primer pairs shown left of the gel images. (**F**) The verification of nonreciprocal type native HRs of 'HANNA' between the A09 and C8 chromosomes by genomic DNA-based real-time PCR. The bar graphs (left) show the relative dosage of A09 genome at each position in 'HANNA' with the dosage in 'CHOY' set at 1. The genome dosage was calculated as the ratio of fluorescent intensity of PCR products obtained by each primer pair to those obtained by the Fw11 & Rv12 primer pair.

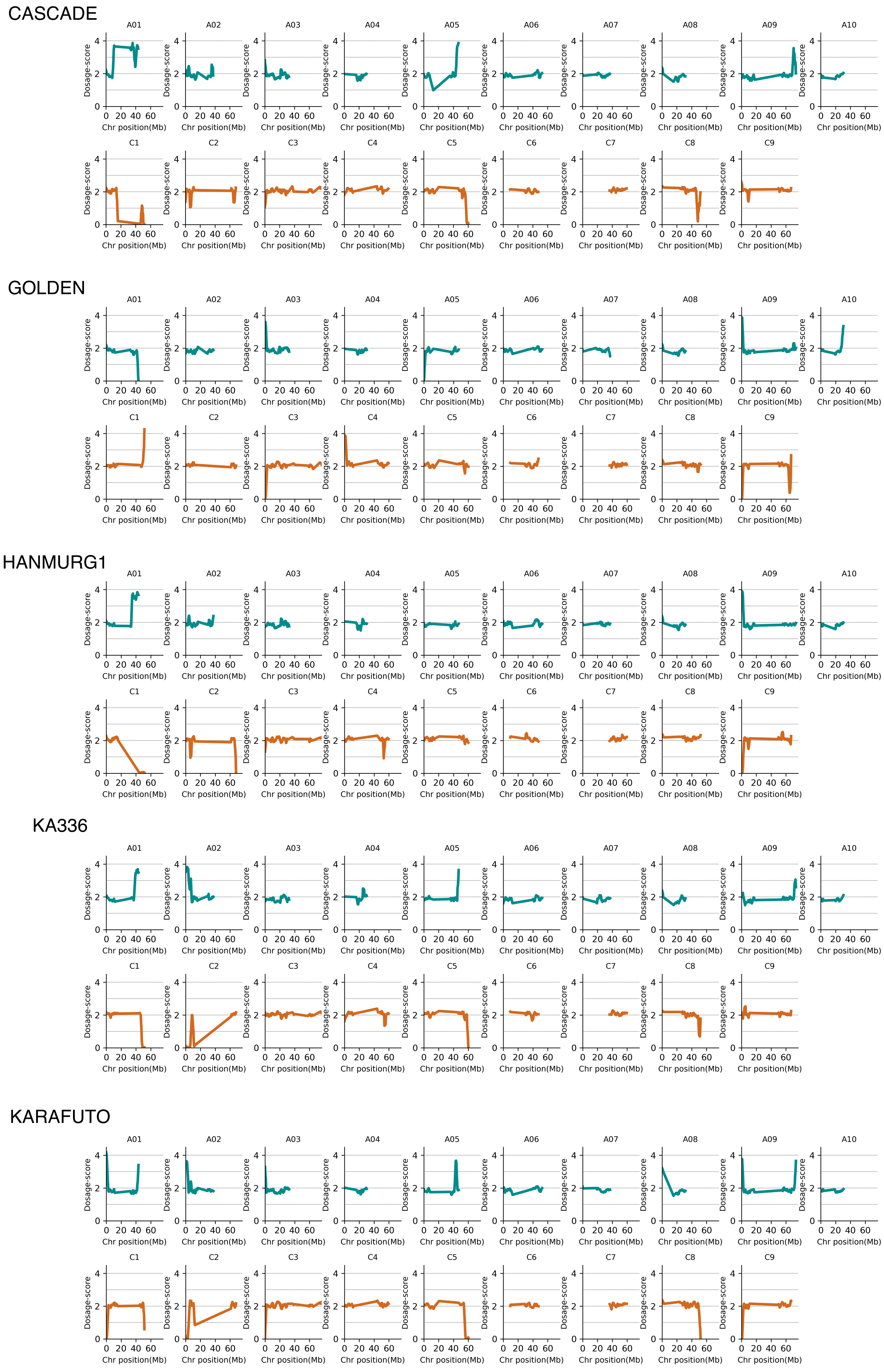
CASCADE
GOLDEN
HANMURG1
KA336
KARAFUTO
A01
A02
A03
A04
A05
A06
A07
A08
A09
A10
C1
C2
C3
C4
C5
C6
C7
C8
C9
Dosage-score
Chr position(Mb)

LAULENTIAN

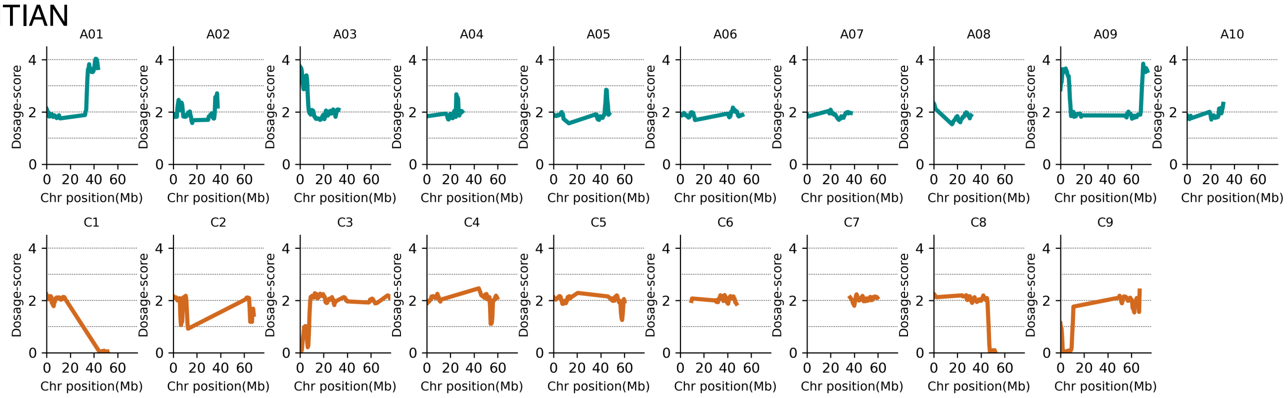

MAGESTIC1

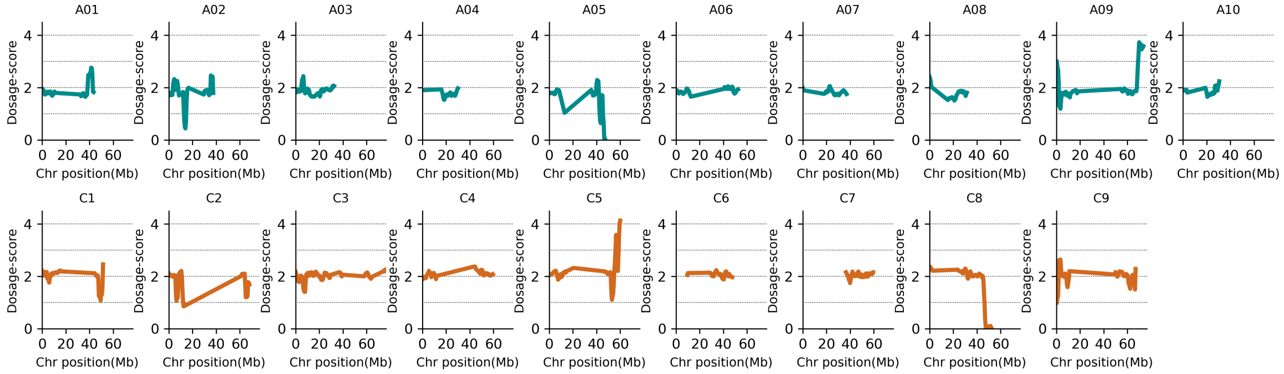

QUINGYOU6

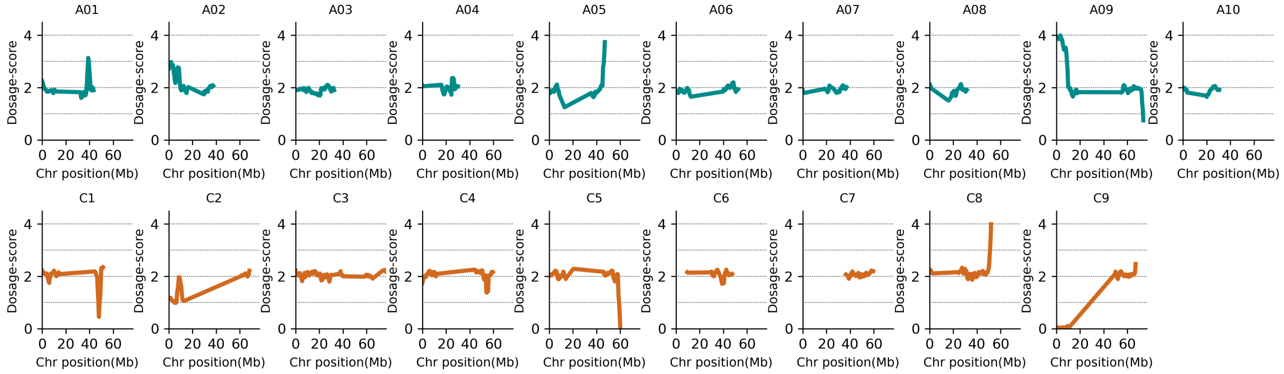

RO6

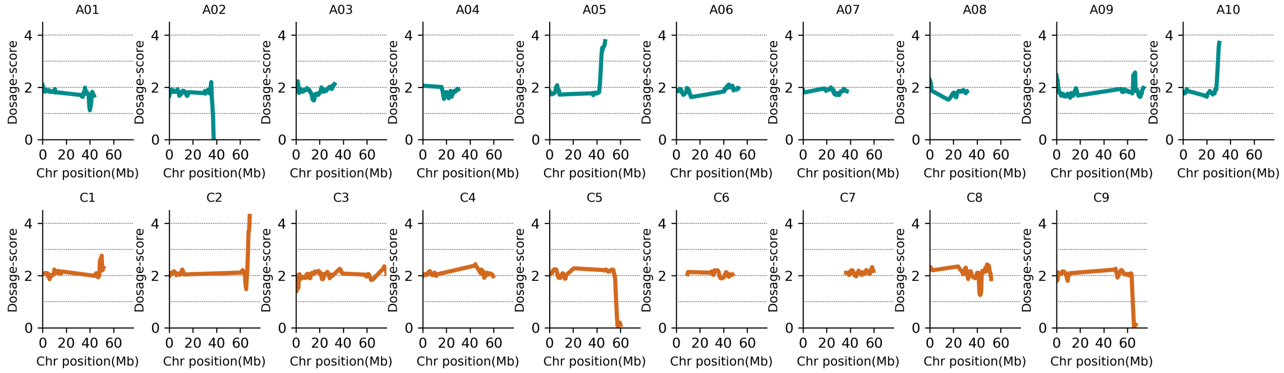

TOPAS

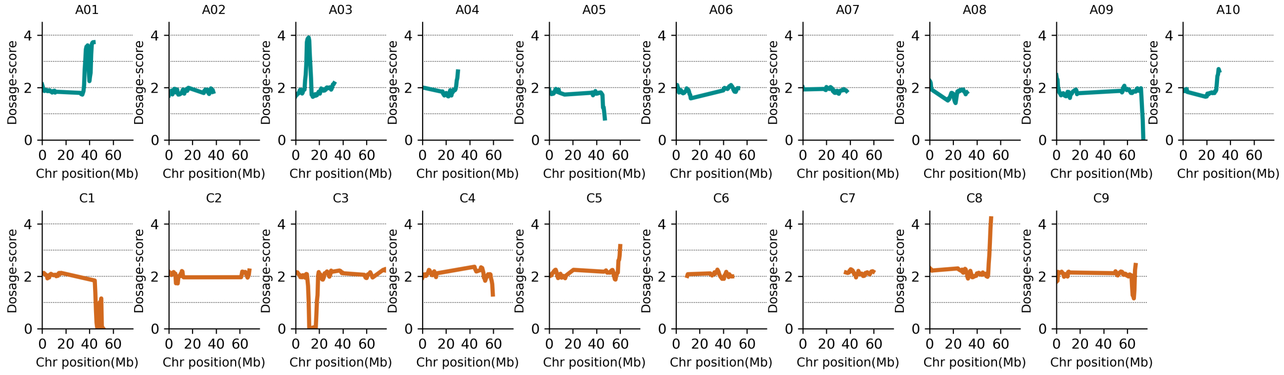

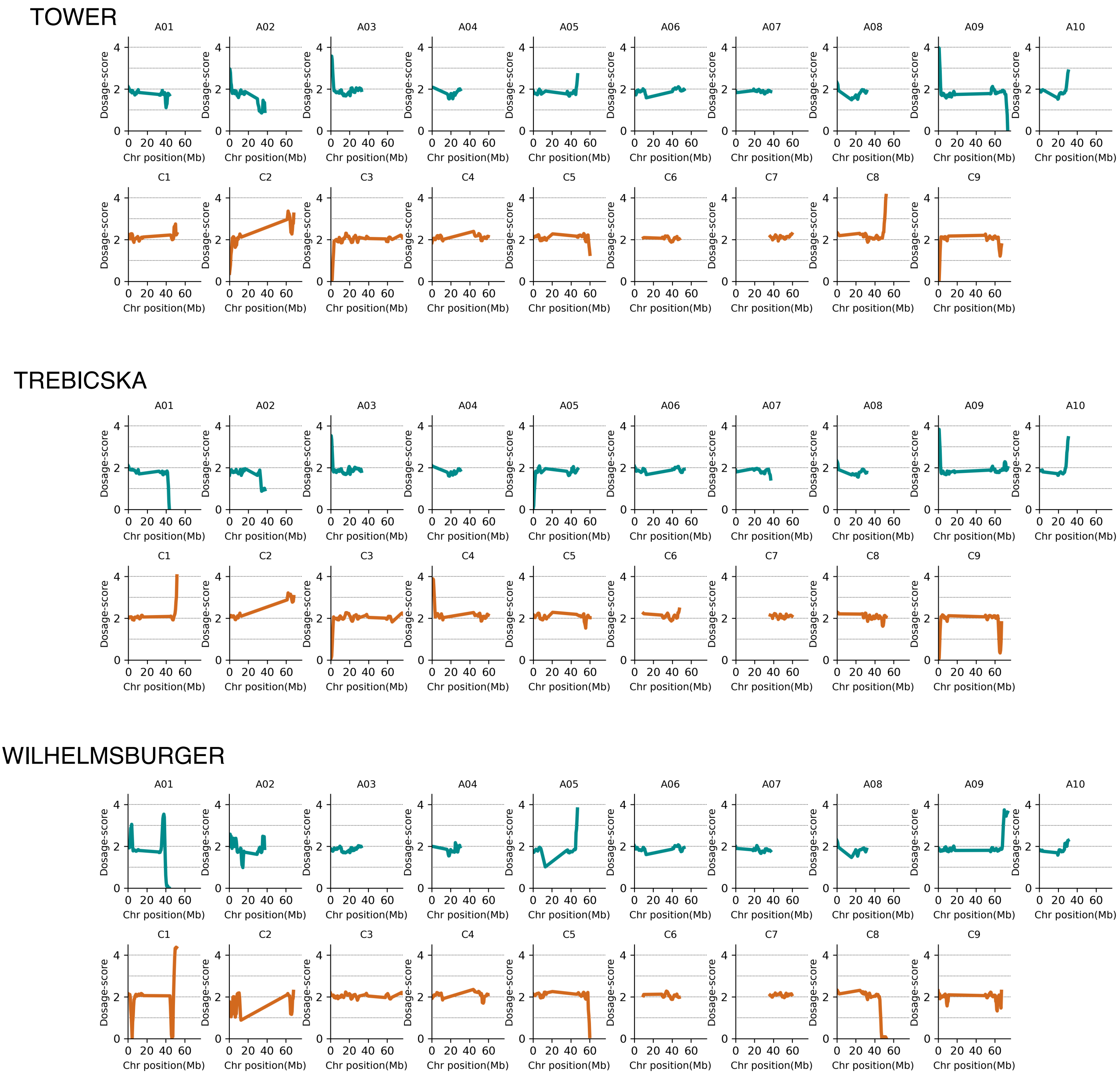

Supplemental Fig. 6. Dosage-score analysis in 13 *B. napus* cultivars.
Dosage-score analysis was set window size=2 Mb and step size=500 kb.

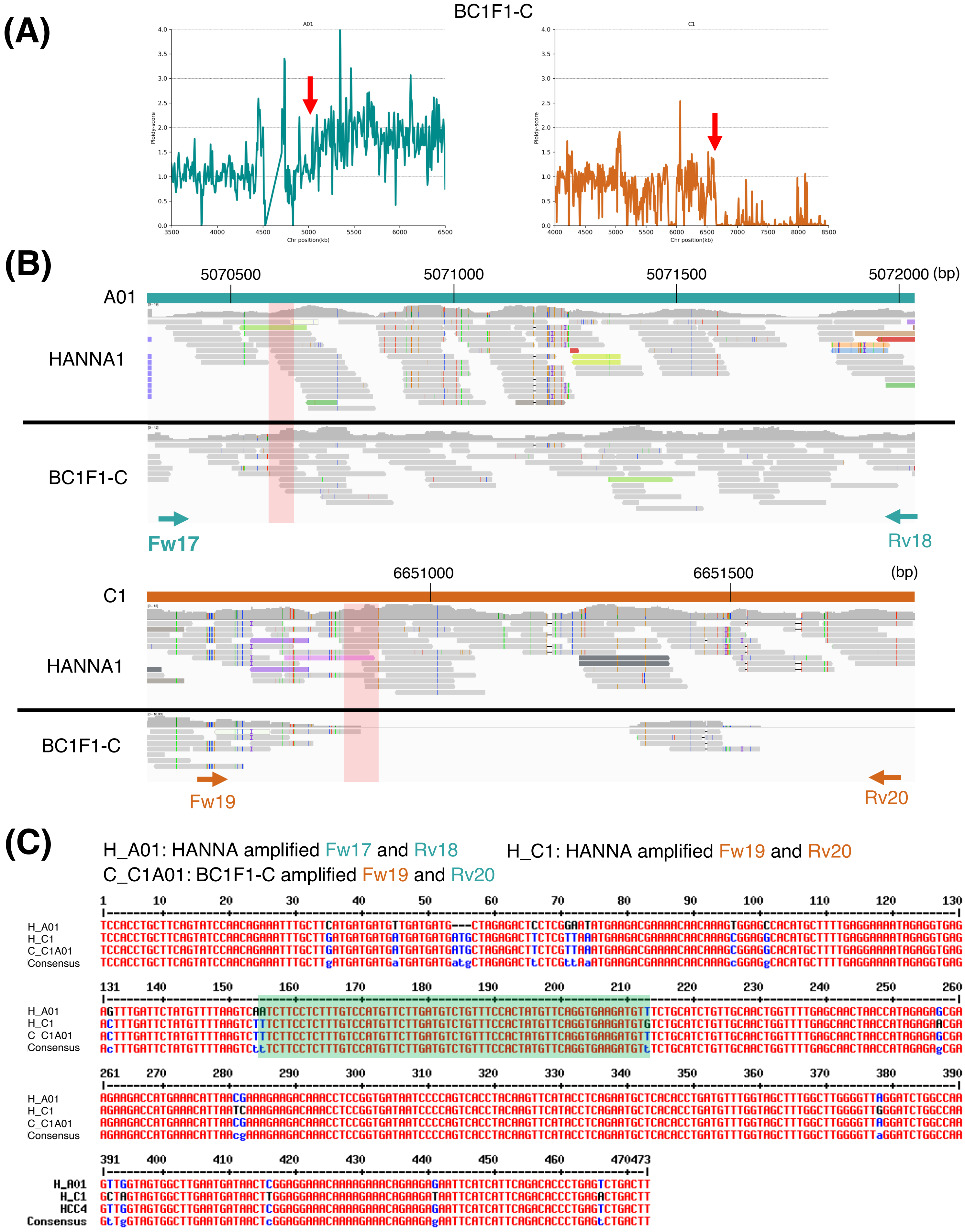

Supplemental Fig. 7. Identifying HR site in A01-C1 that occurred during F1 meiosis.
(**A**) Dosage-scores analysis result obtained for chromosomes A01 and C1 in BC1F1-C using with a sliding window size of 10 kb and step size of 5 kb. Red arrows indicate the positions where significant Dosage-score changes were detected indicating the occurrence of HR. (**B**) IGV images of Illumina short reads aligned to the predicted HR site of the A01 (to panel) and C1 (lower panel) HR regions. Regions shown in red represent the region for which the sequences were confirmed by Sanger sequencing. (**C**) Comparison of sequences obtained by Sanger sequencing of PCR products (Fig. 6A) amplified using primers designed at HR sites in 'HANNA' and BC1F1-C. The region highlighted in red represents the candidate HR site with a resolution of less than 60 bp interval.

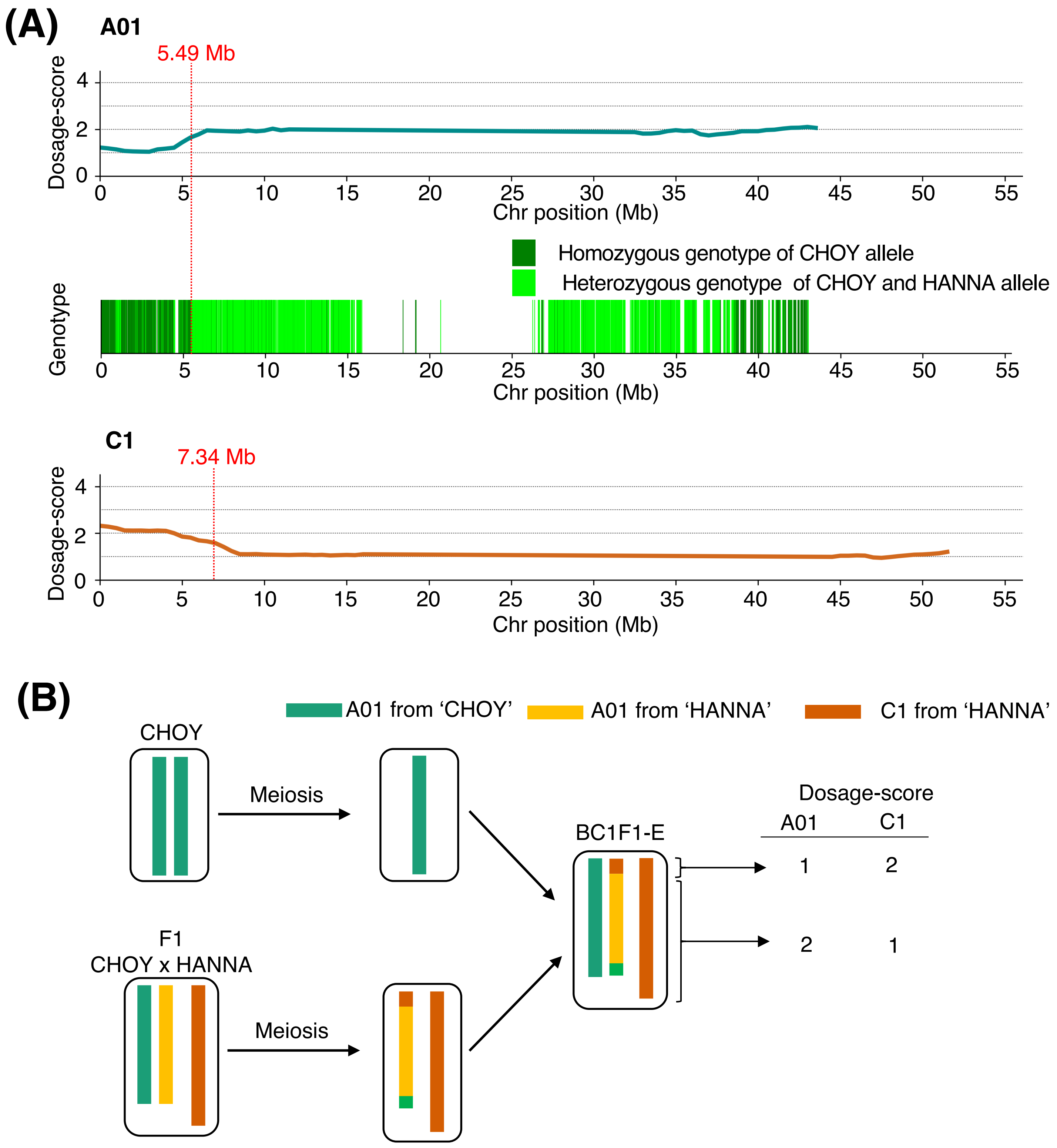

Supplemental Fig. 8. The A01 and C1 chromosome structure of BC1F1-E.
(**A**) The top and bottom panels are the Dosage-score analysis results for A01 and C1 with sliding window size=2 Mb and step size=500 kb. The middle panel is the SNP analysis results for A01. Identification of homozygous and heterozygous regions in BC1F1-E using re-sequencing data and "NapusRef". The positions displaying homozygous SNPs between 'CHOY' and 'HANNA' were detected and included in the subsequent analysis. The positions where the frequency of the 'CHOY' allele was 1 were defined as homozygous for the 'CHOY' allele (depicted in deep green). The positions where the frequency of the 'CHOY' allele ranged between 0.4 and 0.6 were defined as heterozygous genotype (shown in lime green). (**B**) The estimation of meiosis situation from the expected genome structure by Dosage-score and SNP analysis.

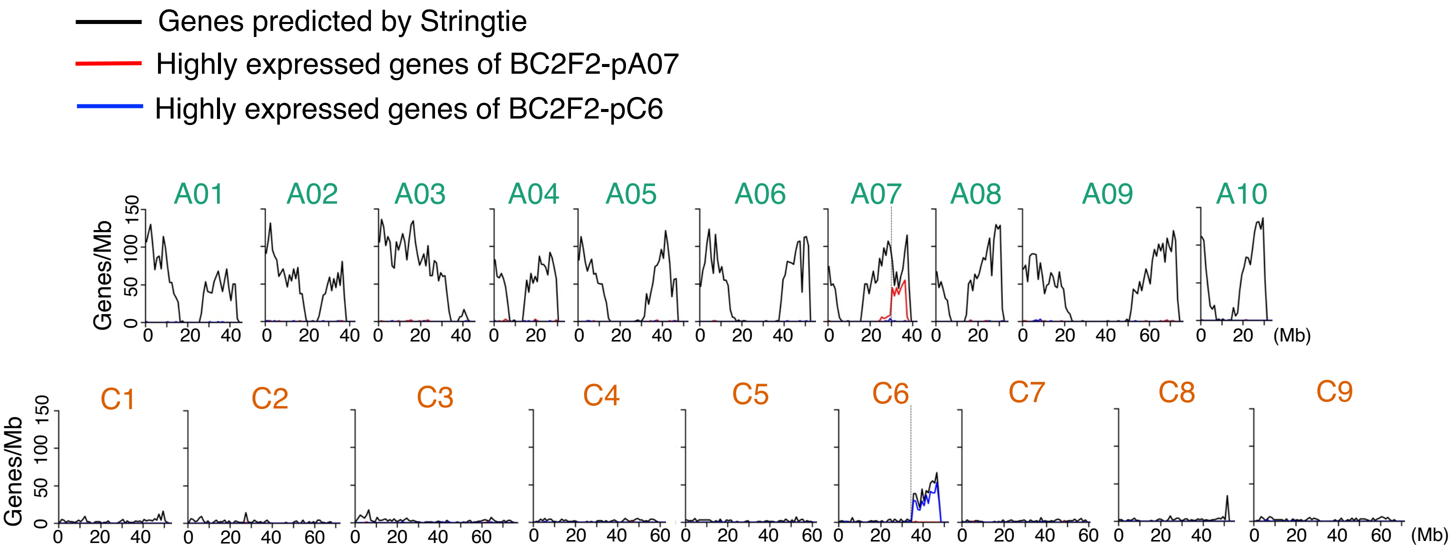

Supplemental Fig. 9. Comparison of the expression patterns of genes in the BC2F2-pA07 and -pC6 genomic regions.

The expression profile of genes showing significant difference between the BC2F2 progeny containing the posterior region of C6 and A07. The *p*-adjusted value in DESeq2 < 0.01 was used define significant differentially expressed genes.
